# Ciliary and cytoskeletal functions of an ancient monooxygenase essential for bioactive amidated peptide synthesis

**DOI:** 10.1007/s00018-019-03065-w

**Published:** 2019-03-16

**Authors:** Dhivya Kumar, Richard E. Mains, Betty A. Eipper, Stephen M. King

**Affiliations:** 1 Department of Molecular Biology and Biophysics, University of Connecticut Health Center, Farmington, CT 06030 USA; 2 Department of Biochemistry and Biophysics, University of California San Francisco, San Francisco, CA 94158 USA; 3 Department of Neuroscience, University of Connecticut Health Center, Farmington, CT 06030 USA

**Keywords:** Actin, Amidation, *Chlamydomonas*, Cilia, Microvilli, Peptidylglycine α-amidating monooxygenase

## Abstract

Many secreted peptides used for cell–cell communication require conversion of a C-terminal glycine to an amide for bioactivity. This reaction is catalyzed only by the integral membrane protein peptidylglycine α-amidating monooxygenase (PAM). PAM has been highly conserved and is found throughout the metazoa; PAM-like sequences are also present in choanoflagellates, filastereans, unicellular and colonial chlorophyte green algae, dinoflagellates and haptophytes. Recent studies have revealed that in addition to playing a key role in peptidergic signaling, PAM also regulates ciliogenesis in vertebrates, planaria and chlorophyte algae, and is required for the stability of actin-based microvilli. Here we briefly introduce the basic principles involved in ciliogenesis, the sequential reactions catalyzed by PAM and the trafficking of PAM through the secretory and endocytic pathways. We then discuss the multi-faceted roles this enzyme plays in the formation and maintenance of cytoskeleton-based cellular protrusions and propose models for how PAM protein and amidating activity might contribute to ciliogenesis. Finally, we consider why some ciliated organisms lack PAM, and discuss the potential ramifications of ciliary localized PAM for the endocrine features commonly observed in patients with ciliopathies.

## Introduction

**Fig. 1:**
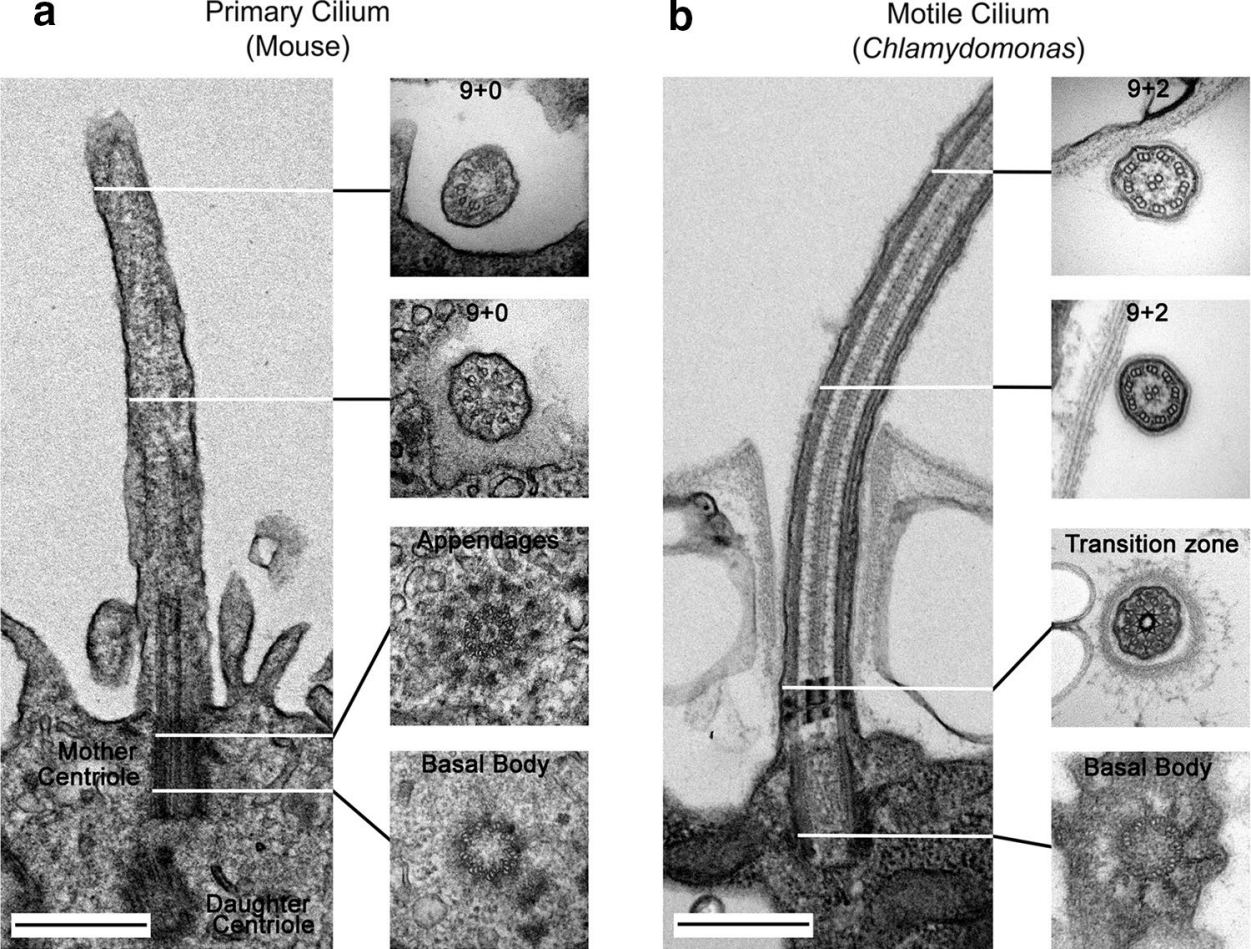
Structure of primary and motile cilia. Electron micrographs showing the basal bodies, transition zones and axonemal structures of an immotile (primary) cilium in the neuroepithelium of an E12 mouse (**a**) and a *Chlamydomonas* motile cilium (**b**). The axonemal microtubules are templated directly by the basal body, a specialized centriole characterized by appendages required for cilium formation. The basal body derives from a mature (mother) centriole while the newly formed (daughter) centriole is oriented orthogonally and located deeper in the cytoplasm (**a**). The primary cilium has a 9 + 0 axoneme consisting only of nine outer doublet microtubules, while most motile cilia have a 9 + 2 structure with an additional central pair microtubule complex involved in motility regulation. The transition zone, which acts as a gate controlling ciliary entry and exit, contains Y-links that connect the membrane to the microtubules. Scale bars = 500 nm. Reproduced from [115] © Dhivya Kumar

Cilia are membrane-bound cellular projections containing a microtubule-based scaffold, the axoneme, that is templated directly from the triplet microtubules of a modified centriole termed the basal body. Cilia are sensory organelles that can be characterized as motile (capable of cell and/or fluid propulsion) or immotile (also known as primary cilia) [1] (Fig. 1a, b). These organelles are of ancient origin, being widely found throughout the eukaryotes and are thought to have been present in the last common ancestor of eukaryotes. They play key roles in monitoring the extracellular environment, processing developmental signals and generating propulsive force and fluid flow [2]. Recent studies suggest that cilia also act as secretory organelles and transduce information in the form of small vesicular packets called ectosomes that play a role in cell–cell communication, intracellular signaling and cell cycle-related processes such as mother cell wall degradation and consequent mitotic progeny release in the unicellular green alga *Chlamydomonas* [3–5].

Nearly all cells in the human body (except those of lymphoid and myeloid origin) build a cilium at some point in their life cycle [6]. In addition to the motile sperm flagellum (a modified cilium), multiple motile cilia are present on the apical surfaces of cells lining various structures. The motile cilia on the ependymal cells lining the ventricles of the brain generate the force needed to move cerebrospinal fluid. In the lung, motile cilia play an essential role in mucus clearance which acts as a first line of defense against airborne pollutants and pathogens. Most other cell types possess a solitary, immotile (primary) cilium (Fig. 1a) that acts as a sensory antenna [7] and in some cases has become highly modified to perform specific tasks, e.g., light detection by the outer segments of rods and cones in the eye and odorant reception by multiciliated olfactory neurons.

Cilia are highly complex: proteomic, transcriptomic and comparative genomics approaches in various organisms have identified many hundreds of proteins associated with these organelles [8, 9]; indeed, a recent estimate suggests that the human “ciliome” consists of approximately 1200 genes [10]. Consequently, perhaps 5% or more of the ~ 21,000 human protein-encoding genes [11] are involved in ciliary assembly, structure and/or function. Cilia are essential for organismal development and homeostasis; defects result in a wide array of ciliopathies [12]—complex syndromes (e.g., Bardet–Biedl [13] and Joubert [14] syndromes) which can have broad phenotypic consequences [15–17].

The cilium is a discrete cellular compartment; entry into both motile and immotile cilia is controlled in part by a multi-subunit gate termed the transition zone [18, 19] (Fig. 1b). Although the ciliary membrane is contiguous with the plasma membrane it has a very distinct lipid and protein content. Numerous receptors/channels are localized to this compartment, allowing the organelle to both sense the extracellular environment and initiate appropriate signaling cascades that relay information to the cell body in response to external chemical or mechanical signals. Well-known primary cilia-dependent pathways include non-canonical Wnt (planar cell polarity) [20, 21] and Hedgehog [22, 23] signaling as well as G-protein coupled receptor-mediated responses to peptide hormones such as somatostatin [24] and kisspeptin [25]. Motile cilia also exhibit sensory functions. For example, ciliated tracheal epithelial cells are mechanosensitive, modulating ciliary beat frequency to match the viscosity of the mucus they encounter [26]. In *Chlamydomonas*, enhanced ciliary power output during viscous loading is mediated by increased trafficking of the dynein regulatory factor Lis1 into cilia [27]. Some cilia also possess chemoreceptors of the bitter taste family that can sense and respond to noxious compounds [28]. In addition, the initial steps of the cAMP-mediated signaling pathway, which are initiated in response to cell–cell contact during sexual reproduction in *Chlamydomonas*, are confined to its motile cilia [29].

Protein and membrane trafficking are critical, yet poorly understood, aspects of cilium assembly. For example, soluble protein components must be specifically moved into the growing cilium, along with new membrane and proteins delivered in Golgi-derived vesicles. Although there are roles for ARF family small GTPases [30] and microtubule motors [31, 32], how the vesicular trafficking process is regulated to control the rate of ciliary membrane addition is uncertain. Ciliary membrane is lost during the budding of ectosomes, a process that involves branched actin filament dynamics [3, 33]. Ciliary integrity and homeostasis require a delicate balance between post-Golgi trafficking and the release of ectosomes. Indeed, ciliary length is a tightly regulated parameter [34] and, for example, can change in response to hypoxia [35].

Recent studies have uncovered an unanticipated role for a secretory pathway enzyme involved in bioactive peptide synthesis (*peptidylglycine α*-*amidating monooxygenase*; PAM) in building cilia [36–38]; this connection has been conserved between *Chlamydomonas* and metazoans (planaria, mice and zebrafish) suggesting that it dates to the last eukaryotic common ancestor and represents an important aspect of ciliogenesis. Here we review the evidence supporting a role for the PAM protein and its amidating activity in ciliary assembly, suggest models for PAM function in this process, and describe how interactions of PAM with the actin cytoskeleton might alter both cilia and microvilli, leading to broad and generalized effects on cytoskeleton-based cellular protrusions. Furthermore, we address the intriguing phylogenetic question of how some organisms that lack PAM can still build cilia and briefly discuss the more clinical implications of merging the fields of ciliogenesis and peptidergic signaling.

## General principles underlying ciliary formation and assembly

**Fig. 2:**
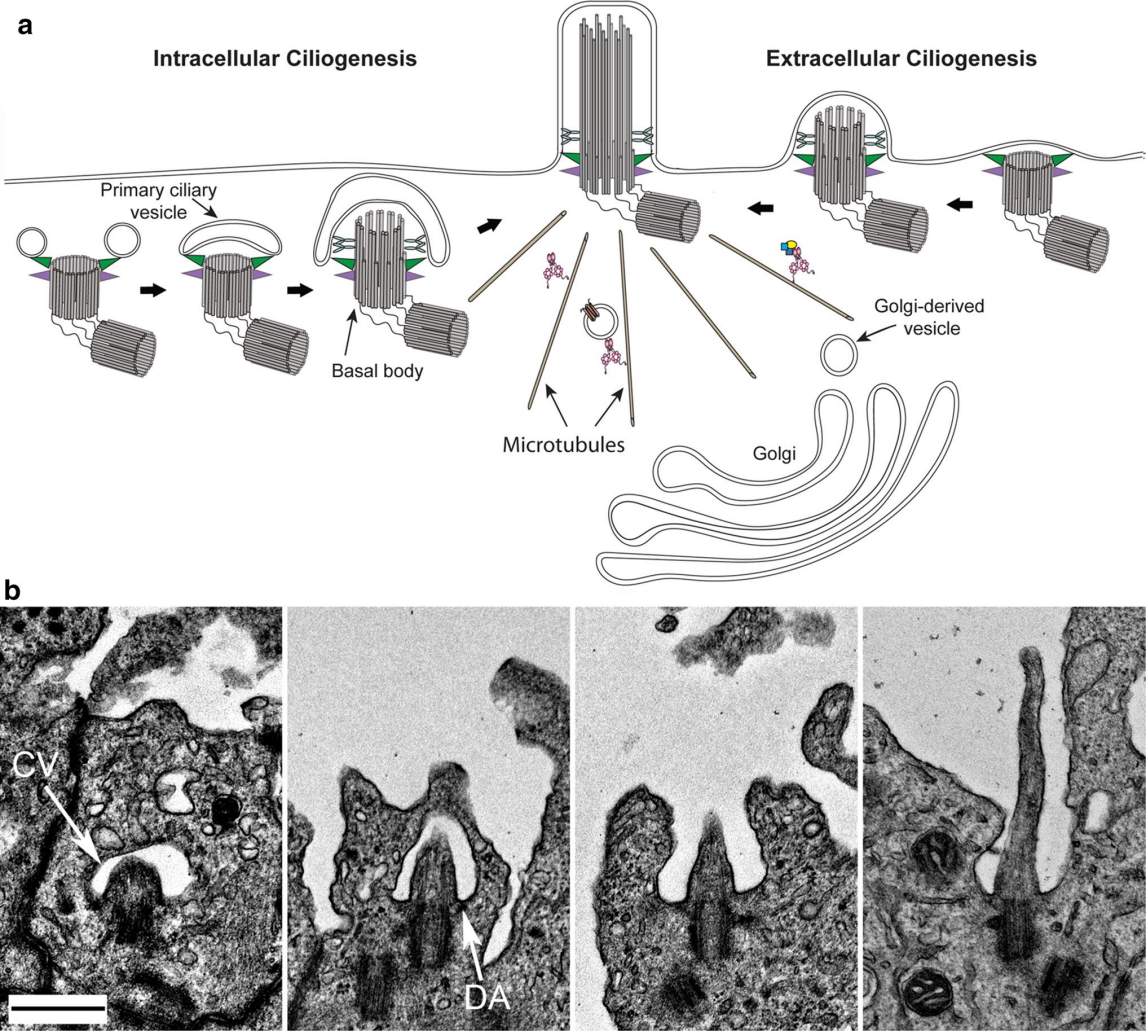
Pathways of ciliogenesis. **a** Schematic showing the two distinct modes of ciliogenesis used by various cell types. Fibroblasts and neuronal progenitors assemble their primary cilium within the cytoplasm prior to docking at the plasma membrane (intracellular ciliogenesis). In contrast, tracheal epithelial cells and *Chlamydomonas* utilize an extracellular pathway (extracellular ciliogenesis) to assemble their motile cilia, which starts with the docking of basal bodies at the membrane. Golgi-derived vesicles, moved along cytoplasmic microtubules by dynein motors, deliver specific membrane proteins and lipids to the growing organelle. Color code: basal body distal appendage, green; basal body sub-distal appendage, purple; transition zone Y-links, blue-gray; dynein, pink; membrane proteins, brown; other dynein cargoes, yellow, blue and light blue. **b** Electron micrographs illustrating the different stages of intracellular ciliogenesis during the assembly of primary cilia in neural epithelial cells from the third ventricle of an E12 mouse. The primary ciliary vesicle (CV) and a basal body distal appendage (DA) are indicated by arrows. Scale bar = 500 nm. Panel (b) is reproduced from [115] © Dhivya Kumar

The process of ciliogenesis varies in different cell types [34, 39, 40] (Fig. 2a). In the extracellular pathway, used by multiciliated epithelial cells and unicellular ciliated organisms such as *Chlamydomonas* and *Tetrahymena*, the basal body (a modified centriole decorated with distal and sub-distal appendages [34]) first docks at the plasma membrane; the axoneme (the microtubule core of the cilium), enveloped by the ciliary membrane, is then extended outwards into the extracellular space. In contrast, other cell types such as fibroblasts and neural progenitors, which build immotile sensory cilia, accomplish many of the initial steps intracellularly (Fig. 2a, b). Initially, small Golgi-derived “distal appendage vesicles” fuse to give rise to a primary ciliary vesicle attached to the distal appendages of the basal body within the cytoplasm (Fig. 2b). Subsequently, the axoneme extends and secondary vesicles fuse with the initial primary ciliary vesicle to accommodate the growing structure. At this stage, the axoneme is covered by two membranes: an inner membrane, which will eventually become the ciliary membrane, and an outer membrane, the sheath, which ultimately fuses with the plasma membrane. After this structure docks at the plasma membrane and fusion occurs, the cilium is extruded and continues to elongate as it is supplied with axonemal building blocks and membrane.

**Fig. 3:**
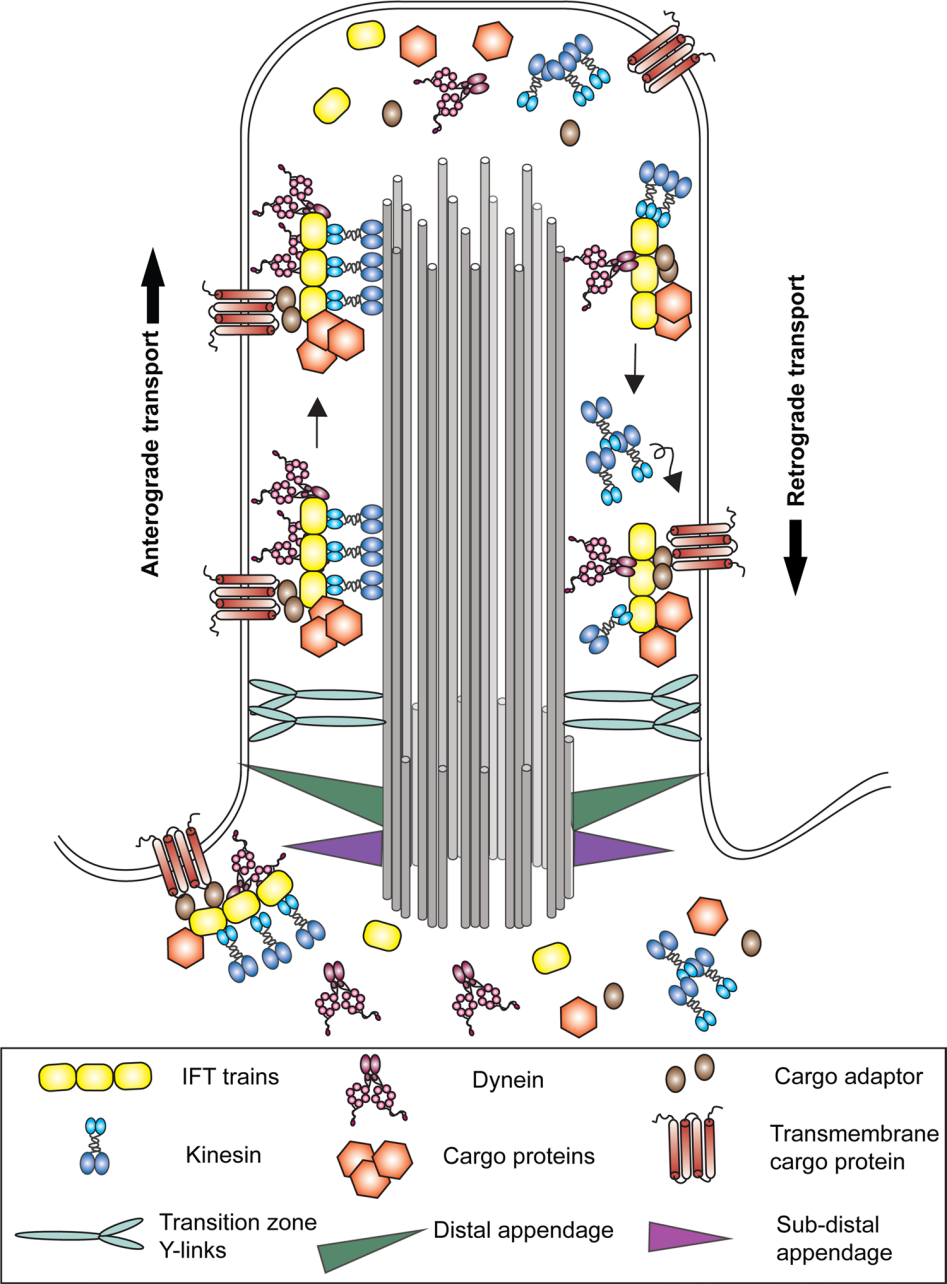
Mechanisms of ciliary trafficking. Diagram illustrating the major systems involved in trafficking components into, within and out of cilia. Intraflagellar transport (IFT) trains with attached axonemal and transmembrane cargo proteins are specifically gated into the organelle at the transition zone and driven towards the ciliary tip by one or more kinesin motors. Following cargo release, remodeling and association of components destined for ciliary export, IFT trains are returned to the cell body by a specific dynein isoform. Note that the anterograde kinesin motor has been proposed to return to the cell body by diffusion in *Chlamydomonas* [45], although in other organisms there is evidence for its return by active dynein-mediated transport [116–118]. Once returned to the cell body, IFT components are thought to disperse into the general cellular pool prior to re-recruitment to the basal body region for reuse [119]. Modified and adapted from [120] under a CCBY license

Since the ciliary matrix lacks ribosomes, proteins are not synthesized in this cellular compartment. Cargoes destined for this organelle must be recognized, sorted and trafficked from the cytoplasm or endomembrane system. This process involves the intraflagellar transport (IFT) machinery that mediates bi-directional transport along ciliary doublet microtubules (Fig. 3). The multimeric IFT-B complex is required for the kinesin-dependent movement of cargo from the ciliary base to the tip [41, 42]. In organisms such as *Chlamydomonas*, the IFT-A complex associates with a specific “cytoplasmic” dynein isoform to return IFT particles to the ciliary base and ultimately to export proteins out of the cilium [43]. In nematodes, the IFT system is somewhat modified and although IFT-A is required for retrograde transport, the IFT-A complex itself has been found to associate with the anterograde heterotrimeric kinesin II motor, while IFT-B is transported by a homodimeric kinesin [44]. In addition to IFT-mediated transport, there is also evidence for retrograde diffusion of certain proteins [45], including α-tubulin and the KIF17 kinesin, within the axonemal lumen of primary cilia [46].

The trafficking of membrane proteins such as PAM into and out of the cilium poses further challenges [47], and their movement is aided by an additional IFT-associated multimeric complex—the BBSome [48–51]. Indeed, current evidence suggests that the BBSome is primarily involved in ciliary protein export [52]. Although some IFT-cargo interactions, including those involved in tubulin and outer arm dynein transport with its associated IFT46 cargo adaptor, are now understood at the structural level e.g., [53, 54], the general principles by which IFT and BBSome complexes recognize the enormous range of ciliary cargoes that must be trafficked remain unclear. Intriguingly, some IFT proteins play non-cilia related roles in the cytoplasm and, for example, have been implicated in cell cycle progression [55], mitotic spindle orientation [56] and cytoplasmic microtubule dynamics [57].

## PAM, bioactivation by amidation and the secretion of signaling peptides

PAM (EC 1.14.17.3) is best known for its role in the neuroendocrine system, where it catalyzes a late step (α-amidation) in the synthesis of many secreted bioactive peptides such as oxytocin, vasopressin, gonadotropin-releasing hormone and neuropeptide Y [58]. As peptide precursors traverse the secretory pathway, they are acted on by proprotein convertases that cleave after paired basic amino acid sites, and then by carboxypeptidases, which remove the basic residues. If during this process a glycine residue is exposed at the C-terminus of the processing intermediate, it becomes a potential substrate for amidation by PAM. Amidation plays three major roles: (1) it alters the charge and structure at the C-terminal end of the peptide allowing specific and high affinity recognition by cognate receptors, (2) it affects overall peptide conformation, and (3) it enhances peptide stability, extending lifetime in the extracellular environment. In addition to their numerous roles in metabolism, tissue homeostasis and other aspects of vertebrate physiology, amidated peptides are commonly used by invertebrates. For example, species-specific amidated peptides released by sea urchin eggs allow for sperm chemoattraction [59], and many of the toxic peptides present in cone snail, bull ant and spider venoms [60–63] are amidated.

**Fig. 4:**
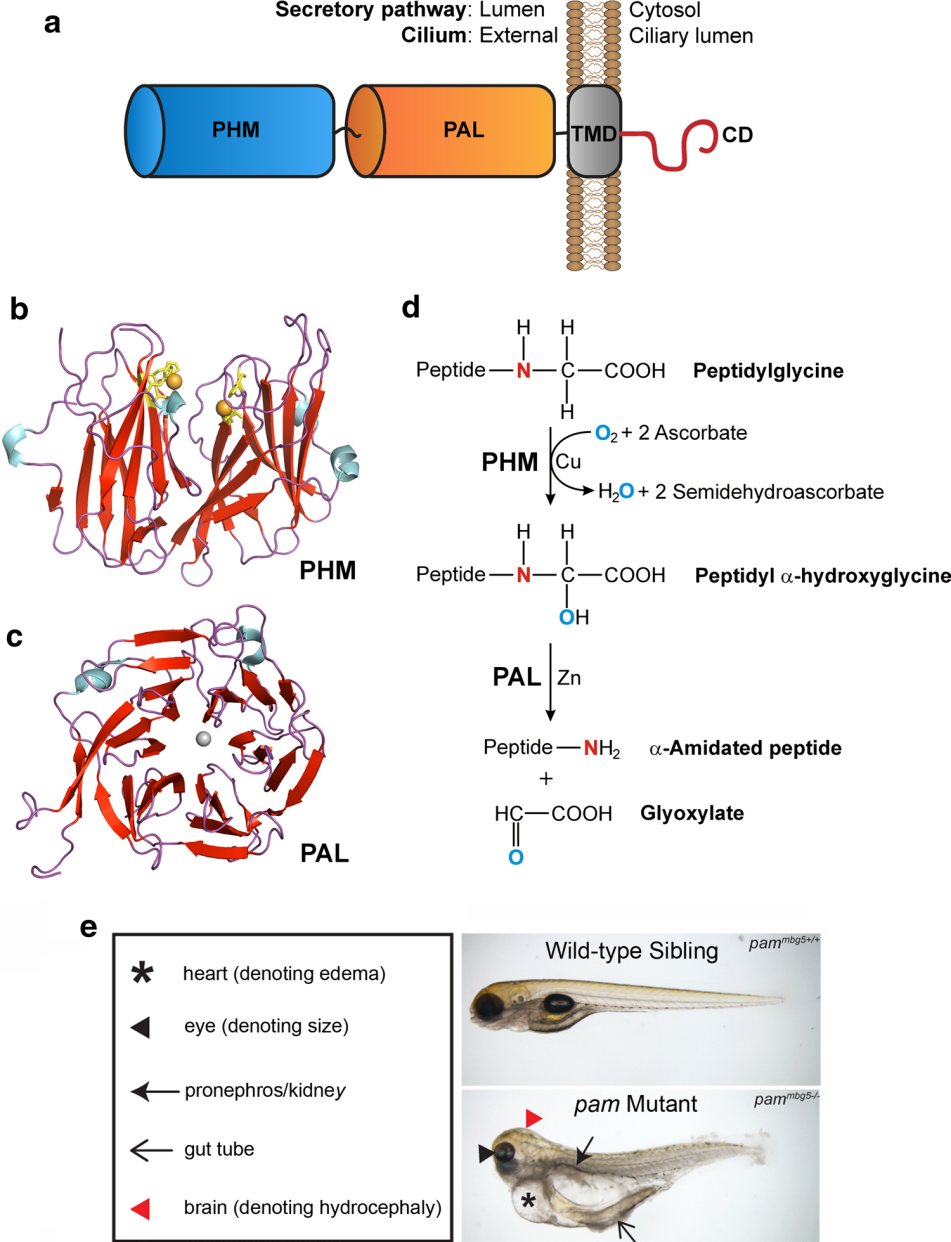
PAM structure, reaction chemistry and zebrafish mutant phenotype. **a** Domain organization of PAM. The two catalytic cores (PHM and PAL) are located in the lumen of the secretory pathway and on the ciliary surface. They are followed by a transmembrane region (TMD) and an unstructured cytosolic domain (CD) that contains signals required for trafficking through the biosynthetic and endocytic pathways [58]. In cilia the CD is located within the organelle, and ciliary *Chlamydomonas* PAM associates with the microtubular axoneme via an unknown mechanism. **b**, **c** Ribbon diagrams of the crystal structures for rat PHM (**b**; 1PHM [121]) and PAL (**c**, 3FVZ [122]) generated using the PyMOL molecular graphics system (Schrödinger, LLC). Both domains are color-coded by secondary structure (red, β-strands; blue, α-helices; purple, loops). The essential Cu atoms (orange) in PHM are coordinated by two His clusters (yellow). PAL forms a six-bladed β-propeller with a single Zn atom (grey) at the active site. **d** Mechanism of the amidation reaction. Glycine-extended peptide precursors are stereo-specifically hydroxylated on the Cα carbon by PHM in a process that requires copper bound at two different sites, molecular oxygen and two molecules of ascorbate [121]. Subsequently, the Cα-N bond is cleaved by PAL to yield the amidated peptide and release glyoxylate. **e***Pam*-null zebrafish embryo and wild-type sibling at 5 days post-fertilization (dpf) [38]. The mutant exhibits pericardial and abdominal edema, hydrocephalus and small eyes; these embryos die at ~ 10 dpf. Panel (e) is modified from [38] under a Creative Commons license

PAM is a Type 1 integral membrane protein with two catalytic cores located in the lumen of the secretory pathway, followed by a transmembrane domain and a cytosolic C-terminal region that is not required for catalysis but is necessary for trafficking the enzyme through the biosynthetic and endocytic pathways (Fig. 4a). PAM produces amidated peptides from glycine-extended precursors in a two-step process. Initially, the *peptidylglycine α*-*hydroxylating monooxygenase* (PHM; Fig. 4b) domain catalyzes the copper-, oxygen- and ascorbate-dependent hydroxylation of the α-carbon of the C-terminal glycine. This is followed by cleavage of the Cα-N bond by the *peptidyl*-*α*-*hydroxyglycine α*-*amidating lyase* (PAL; Fig. 4c) domain, a zinc-dependent enzyme, to release glyoxylate and the α-amidated peptide product (Fig. 4d). Although PAL activity is required in the acidic environment of the secretory pathway, this second reaction step can occur spontaneously as pH rises above neutrality.

PAM is essential for vertebrate development and edema is a major feature observed in mice and zebrafish lacking PAM. *Pam*-null mice die by embryonic day (E) 14.5 with ventricular hypertrophy, massive edema and a poorly formed vasculature [64]. Homozygous *pam*^−*/*−^ zebrafish embryos exhibit small eyes, cyst-like protrusions associated with the pronephros, hydrocephalus and edema, dying at ~ 10 days post-fertilization (Fig. 4e) [38]. PAM is highly expressed in the mouse heart at these early developmental stages but is not highly expressed in zebrafish heart. Nevertheless, in both cases the edema is first observed in the pericardial region and likely derives from altered hormonal signaling and consequent alterations in fluid homeostasis. In *Drosophila*, which has separate *dPHM* and *dPAL* genes, loss of the *dPHM* gene, which encodes soluble amidating enzyme, leads to larval lethality during molting due to defective peptidergic signaling [65].

It is now clear that bifunctional, integral membrane PAM predates evolution of the nervous system [66]. A PAM-like gene is present throughout the metazoa including sponges and placozoans that lack neurons; insects are an exception as they lack integral membrane PAM but rather express separate PHM and PAL proteins. Bifunctional PAM is also found in unicellular and colonial chlorophyte green algae (*Chlamydomonas*, *Gonium* and *Volvox*), strongly suggesting that this enzyme was present in the last eukaryotic common ancestor which is thought to have had a motile cilium that was also used for signaling [36, 66].

## Post-Golgi trafficking, ciliary/cellular localization and topology of PAM

**Fig. 5:**
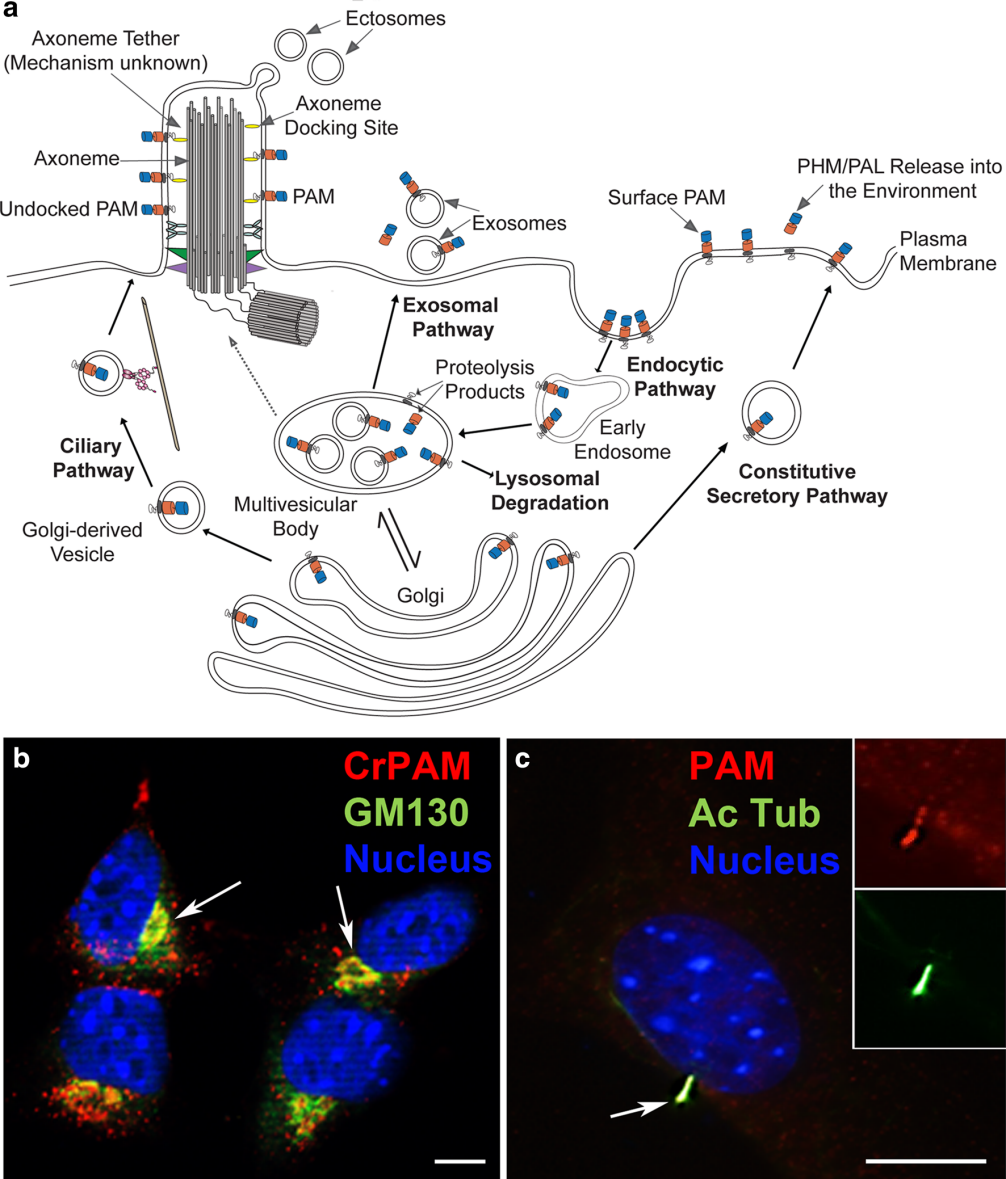
PAM trafficking pathways. **a** Diagram illustrating the pathways that may be taken by PAM as it moves out of the Golgi complex and through the endomembrane system. PAM contained in constitutive secretory pathway vesicles (the regulated secretory pathway is not shown) appears on the plasma membrane following exocytosis. The active catalytic domains can be shed following proteolysis, but most PAM is rapidly endocytosed, leaving only a small fraction of total cellular PAM (~ 2%) on the cell surface at steady state. In mammalian cells, PAM appears in early endosomes before it is found on the limiting membrane of multi-vesicular bodies. Consistent with its identification in exosomes, PAM can enter the intraluminal vesicles contained within multi-vesicular bodies; fusion of the multi-vesicular body with the plasma membrane results in release of exosomes. Proteolytic cleavages that separate the catalytic cores from the TMD and CD of PAM occur in the multi-vesicular body, allowing release of soluble PAM and generation of sfCD. The factors governing recycling versus lysosomal degradation of PAM are poorly understood. PAM may enter Golgi-derived vesicles that transport specific lipid and protein cargoes to the cilium (ciliary pathway). Alternatively, ciliary PAM may derive from the endocytic pathway (dashed line). Within cilia, the PAM catalytic domains are on the external surface. Like polycystin 2, PAM associates with the axoneme via an unknown mechanism. **b** Murine AtT20 corticotrope tumor cell expressing *Chlamydomonas* PAM. Stably transfected cells were stained to reveal CrPAM (red), GM130 (a Golgi marker; green) and the nucleus (blue). PAM is present in the Golgi complex (white arrows) and numerous small vesicles throughout the cytoplasm [36]. **c** Optical section of a serum-starved NIH 3T3 cell stained for endogenous PAM (red), acetylated tubulin (green) and the nucleus (blue). PAM co-localized with acetylated tubulin within the primary cilium (white arrow) [36]. Scale bars = 10 μm. Panels (b) and (c) were adapted from [36] under a Company of Biologists publication agreement

In mammalian cells, PAM is subject to complex, cell type-specific trafficking through the biosynthetic and endocytic pathways [67]; the trafficking of PAM to mammalian cilia has not yet been examined (Fig. 5a–c). At steady state, very little PAM is found on the plasma membrane; nevertheless, surface biotinylation and antibody internalization studies revealed a significant flux of PAM protein onto and off of the plasma membrane. Newly synthesized PAM that exits the trans-Golgi can enter the constitutive or regulatory secretory pathway. Following exocytosis, PAM appears briefly on the plasma membrane. While plasma membrane-bound or extracellular proteases can release its catalytic cores into the extracellular environment, most plasma membrane PAM undergoes clathrin-mediated endocytosis, after which it can be recycled to secretory granules, cleaved by γ-secretase or degraded. Endocytosed PAM appears rapidly on the external membranes of maturing multivesicular bodies (MVBs). Its entry into the intraluminal vesicles (ILVs) that characterize MVBs is a temperature-dependent step controlled in part by the phosphorylation state of its cytosolic domain (CD). Fusion of MVBs with the plasma membrane leads to the release of ILVs (exosomes); consistent with the presence of PAM in ILVs, PAM has been identified in both urinary and salivary exosomes [68, 69].

Separation of the catalytic domains of PAM from its TMD/CD allows intramembrane proteolysis by γ-secretase, producing a soluble cytosolic fragment (sfCD) that traffics to the nucleus and alters the expression of a subset of genes [70, 71]. Golgi-derived vesicles destined for cilia are known to transport both ciliary membrane proteins and specific lipids [72, 73], and potentially contain PAM (Fig. 5a). Alternatively, ciliary PAM may derive from endosomes recycling PAM from the cell surface; directed exocytosis would allow internalized PAM to enter the cilium, as has been proposed for the Kim1 protein [74, 75]. Indeed, in *Chlamydomonas*, ~ 7% of the total PHM activity is present in cilia [36]. Disrupting the Golgi with Brefeldin A leads to cell body accumulation of PAM and Arf1, a vesicle trafficking factor, and to a decrease in the modified tubulins normally present in *Chlamydomonas* cilia. Similarly, *Chlamydomonas* PAM lacking most of its CD exhibits impaired trafficking; although the mutant protein is still found in the cilia, it tends to accumulate in the secretory pathway [37]. The trafficking determinants in the CD appear to have been well conserved as *Chlamydomonas* PAM is distributed appropriately when expressed in murine cells (Fig. 5b).

Given PAM’s topology in the secretory pathway, the catalytic domains are predicted to be on the outside of the cilium (Fig. 5a) where they could potentially act catalytically on soluble glycine-extended factors in the environment; i.e., locally generating amidated products on/near the ciliary surface. This domain orientation was directly demonstrated in *Chlamydomonas* cilia using antibodies against both the PAM luminal domain and the CD; the latter only yielded a signal after the ciliary membrane had been permeabilized [36]. Intriguingly, immuno-electron microscopy revealed that PAM staining exhibits a distinct periodicity of ~ 250 to 300 nm along the *Chlamydomonas* cilium, and biochemical fractionation demonstrated that PAM associates with the microtubular axoneme as does another ciliary membrane protein—the non-selective cation channel, polycystin 2 [76]; whether this tethering occurs directly through the PAM CD or via some other axoneme-associated component remains uncertain.

## PAM-actin associations and microvillus formation

**Fig. 6:**
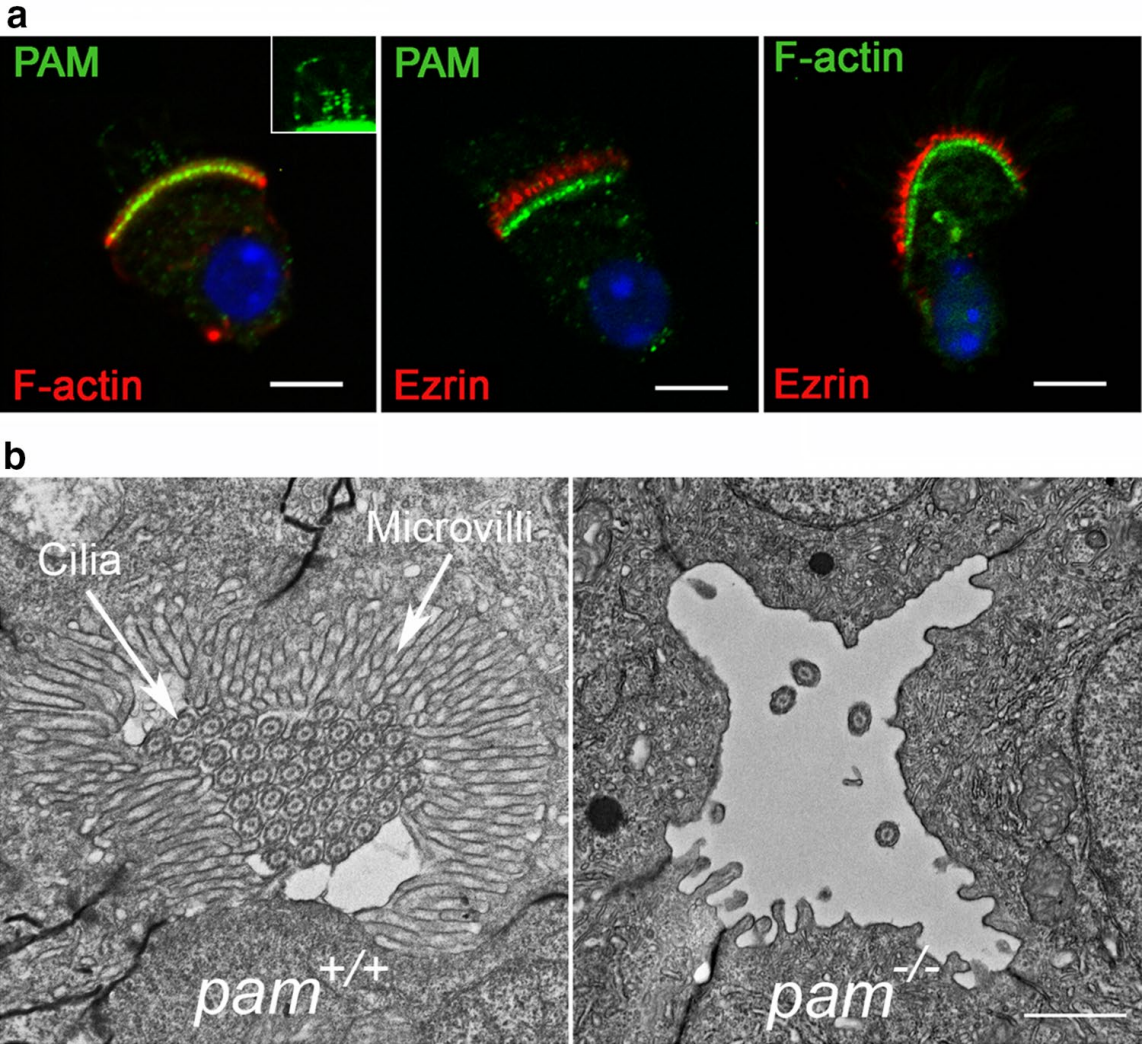
PAM-actin associations and microvillus formation. **a** Immunofluorescence images of murine tracheal epithelial cells illustrating that PAM (green) and F-actin (as detected by fluorescent phalloidin) colocalize in the peri-basal body region, whereas ezrin (red), which marks the microvilli, is more distal; note that microvillar actin is not strongly stained by fluorescent phalloidin presumably because the binding sites are occluded by other microvillar components. The inset (brightness/contrast-adjusted) illustrates the PAM-positive puncta in cilia. **b** Electron micrographs of transverse sections through the pronephros of wild-type and *pam*^−/−^ homozygous mutant zebrafish at 6 days post-fertilization. In the controls (*pam*^+*/*+^), a dense array of microvilli surrounds the numerous closely packed cilia in the pronephric lumen. In contrast, lack of PAM results in complete loss of microvilli and most cilia within the pronephros of the mutant (*pam*^−*/*−^). Scale bars = 5 μm and 1 μm in **a** and **b**, respectively. Modified from [38] under a Creative Commons license

The actin cytoskeleton is intimately involved in the formation of cilia [77], has effects on vesicular trafficking and transcriptional regulation [33], is important for ectosome release during receptor-mediated ciliary signaling [3], and participates in ciliary tip excision [78]. Numerous studies point to a key role for PAM in organizing and controlling the actin cytoskeleton. For example, overexpression of PAM in AtT20 corticotrope cells leads to a dramatic reorganization of cytoplasmic actin resulting in a mainly cortical array and disrupts regulated secretion [79]. In mammals, the PAM CD associates with Kalirin and Trio, modular proteins containing a series of spectrin-like repeats, two guanine exchange factor (GEF) domains, an SH3 domain and a C-terminal kinase module [80]. These GEFs activate Rac1, RhoG and RhoA, which in turn regulate actin filament assembly and dynamics [81]. Furthermore, rat PAM CD also binds actin directly with sub-μM affinity [38]. On its own, the PAM CD does not alter filament assembly dynamics in standard pyrene-actin assembly/disassembly assays. However, as PAM and small vesicular structures colocalize with a dense actin array within the peri-basal body region of multiciliated tracheal epithelial cells, the CD may aid in tethering PAM-containing vesicles to the actin cytoskeleton (Fig. 6a). In *Chlamydomonas*, lack of PAM results in upregulation of an actin paralogue that can functionally compensate for the loss of canonical actin in essential cellular systems; upregulation of this paralogue also occurs in the *ida5* actin-null mutant [82], so in this particular phenotypic response, loss of PAM in *Chlamydomonas* mimics loss of actin. Furthermore, in *Chlamydomonas* cells lacking PAM, cellular actin is reorganized from a diffuse array spread throughout the cytoplasm into a few phalloidin-stained foci [38].

Further evidence that PAM plays a key role in the formation and/or maintenance of actin-based structures came from observations in mutant zebrafish [38] (Fig. 6b). In wild-type embryos at 3 days post-fertilization, the lumen of the pronephros (kidney) is almost completely filled by motile cilia surrounded by a dense outer array of microvilli. In zebrafish, many mRNAs are maternally loaded into the early zygote and indeed *Pam* mRNA is one of the most abundant [83]. Initially, both cilia and microvilli form in *pam*-null mutant embryos as PAM protein can be made at very early developmental stages from the large maternally derived mRNA stores. However, after several days of development, there is no detectable amidating activity and a dramatic loss of microvilli and motile cilia is observed along almost the entire length of the pronephros, suggesting that these structures either cannot form or be maintained in the absence of PAM. As PAM is absent from the microvilli themselves (Fig. 6a), the essential role this enzyme plays in their assembly and/or maintenance must be indirect.

Thus, although the observed changes are both complex and varied, and the vertebrate and *Chlamydomonas* PAM-CD sequences are highly divergent, control of actin cytoskeletal dynamics and behavior is a fundamental property of PAM that has been highly conserved across the eukaryotes.

## PAM and amidating activity are needed to build cilia

**Fig. 7:**
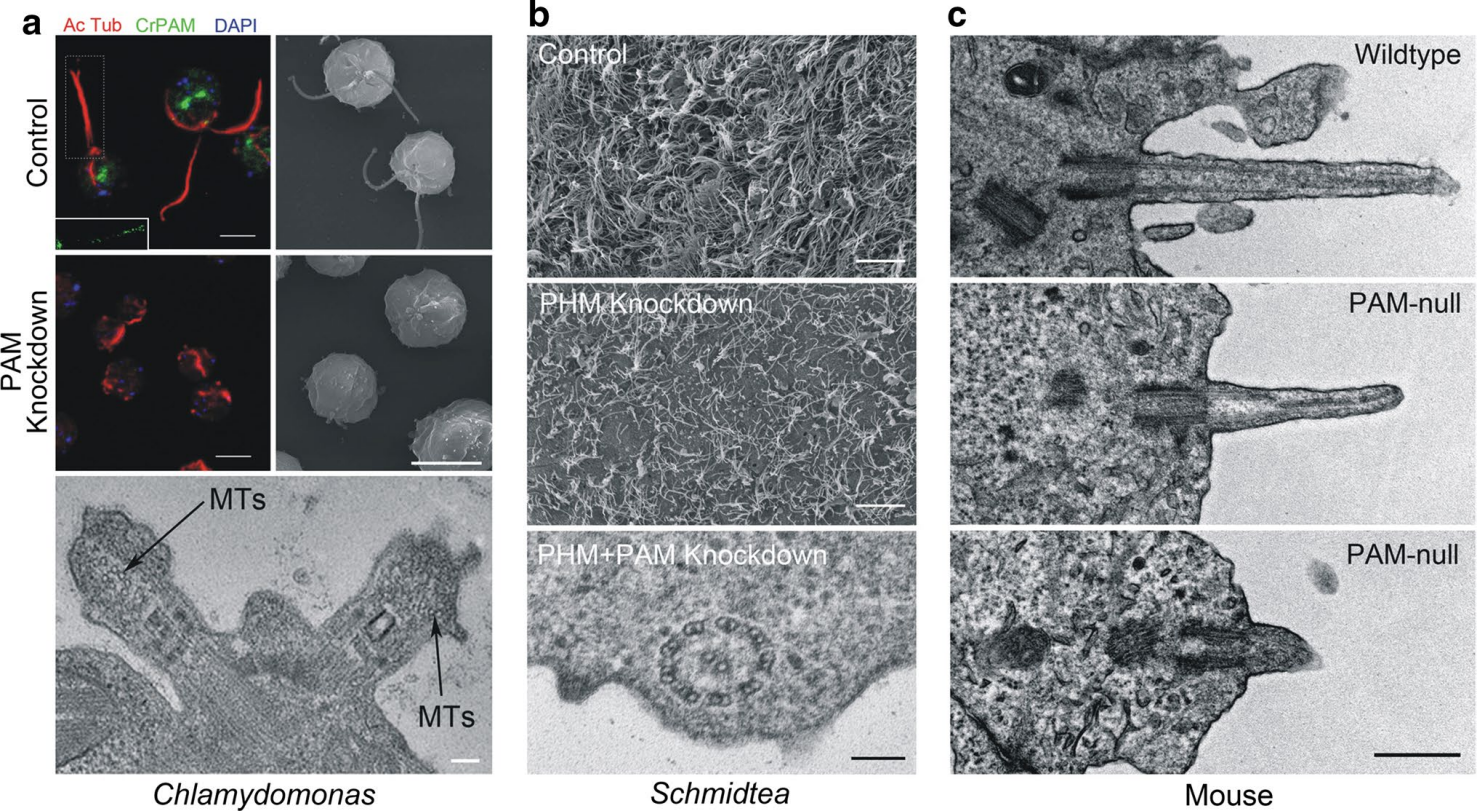
PAM plays a role in ciliogenesis in *Chlamydomonas*, planaria and mice. **a** Immunofluorescence (red, acetylated tubulin; green, PAM; blue, DNA) and scanning electron micrographs of control and PAM knockdown *Chlamydomonas* cells (upper panels; scale bars = 5 μm). Lack of PAM results in the failure of ciliary assembly and the formation of short ciliary stubs that accumulate IFT material and short microtubule fragments (lower panel; scale bar = 100 nm). The inset shows the PAM staining in the cilia (boxed) of the left-most cell—the ciliary tip is oriented to the right. **b** Scanning electron micrographs of the ventral surface of control and PHM-knockdown planaria (upper and center panels; scale bars = 10 μm); reducing PHM activity leads to the loss of cilia as they fail during remodeling. The lower panel illustrates the presence of a morphologically normal axoneme lacking a ciliary membrane in the cytoplasm of a ciliated epithelial cell from a PHM + PAM knockdown planarian (scale bar = 100 nm). **c** Electron micrographs of primary cilia on the neuroepithelium of wild-type and PAM-null mice at E12.5 (scale bar = 500 nm). Modified from [37] under a Creative Commons license

Reducing PAM levels to ~ 10% of wild-type in *Chlamydomonas* revealed an unexpected phenotype [37]. Specifically, PAM-deficient cells were completely unable to build cilia, and instead assembled only short ciliary stubs containing accumulated IFT proteins and short singlet microtubule fragments oriented approximately orthogonal to the normal ciliary long axis (Fig. 7a). Analysis of the transition zone (TZ) in PAM-deficient cells revealed the absence of normal Y-links that form part of the ciliary gate [84, 85]. Although levels of several ciliary proteins were unaltered in PAM-deficient cells, there was a striking increase in the amounts of two TZ proteins (NPHP4 and CEP290) and one IFT-A protein (IFT139), presumably as part of a cellular response attempting to repair or overcome detected abnormalities in *Chlamydomonas* ciliogenesis. These observations suggest that in the absence of adequate amounts of PAM, there is a defect in either the IFT process itself, or in the ability of IFT particles to exit the organelle through the malformed TZ gate.

Golgi morphology was altered in these knockdown strains, as the stacks were more curved than in controls, possibly due to the loss of PAM-actin interactions. PAM deficiency also reduced trafficking of Golgi-derived starch metabolic enzymes, leading to changes in starch granule size, and there were defects in both basal and nutrient deprivation-stimulated secretion. Importantly, PAM knockdown strains grew at the same rate as controls (under both photoautotrophic and photoheterotrophic conditions), and exhibited a normal contractile vacuole cycle, which is another process heavily dependent on membrane trafficking. Thus, the ciliary and Golgi defects observed following the loss of *Chlamydomonas* PAM derive from the disruption of specific cellular processes and do not merely reflect a general decrease in cell fitness or viability.

Demonstration that PAM plays a role in metazoan ciliogenesis came from studies in the planarian *Schmidtea mediterranea* and two vertebrates (zebrafish and mice) [37, 38]. Planaria have a ventral ciliated epithelium used for gliding locomotion and express three PAM-related proteins: a canonical bifunctional integral membrane PAM as well as separate, soluble PHM and PAL proteins that would reside within the secretory pathway. Knock down of membrane PAM and soluble PHM together, using RNAi constructs, reduced enzyme activity to < 10% of control levels and dramatically reduced the number of motile cilia on the ventral surface (Fig. 7b). The remaining cilia were dyskinetic and often had aberrant axonemal architecture likely due to defective remodeling. The double knockdown animals moved at a much slower rate than controls, consistent with movement driven by contractions of the body musculature rather than ciliary beating against secreted mucus. Analysis of the ciliated epithelial cells in these knockdown animals revealed some basal bodies docked at the plasma membrane lacking axonemal extensions, and numerous morphologically normal motile ciliary axonemes located in the cytoplasm very close to the plasma membrane but with no surrounding ciliary membrane (Fig. 7b, lower panel). Strikingly, these unusual cytosolic axonemes were in general oriented with their distal end towards the head of the animal. This latter observation suggests that lack of PAM affects the docking of at least some basal bodies and/or trafficking of Golgi-derived ciliary membrane components. Intriguingly, this “cytosolic axoneme assembly” phenotype was also seen in cells lining the pronephros of *pam*-null zebrafish [38], suggesting that it is a conserved response of multiciliated cells to the lack of PAM.

In mice, lack of PAM is lethal in early development, prior to the formation of multiciliated epithelial cells [64]. However, primary cilia in the developing neuroepithelium of *Pam*^−*/*−^ embryos were much shorter than controls (0.5 versus 0.9 μm), suggesting that they represent immature or aberrant forms (Fig. 7c). Zebrafish *pam*^−/−^ mutant embryos have several cilia-related phenotypes, including small eyes, kidney-associated cysts and hydrocephalus [86], and exhibit loss of both cilia and microvilli in the pronephros within 5 days after fertilization [38]. Interestingly, some ciliary structures, including those in the olfactory bulb, otic vesicles and on neuromasts, appear generally unaffected.

A key question raised by these observations is whether the ciliary phenotypes are caused by the lack of PAM-mediated protein–protein interactions, by the loss of amidating activity per se, or both. Currently, several lines of evidence provide support for a role for amidating activity. First, planaria exhibited the strongest ciliogenesis phenotype only when both membrane-PAM and soluble PHM were targeted together, thereby reducing amidating activity to very low levels. Furthermore, knockdown of soluble PHM alone resulted in ciliary loss and the slow muscle-driven motility characteristic of animals with defective motile cilia [87], again suggesting a role for monooxygenase activity. Second, PHM is inhibited by 4-phenyl-3-butenoic acid (PBA) [88], a suicide inhibitor, and by neocuproine, a copper-specific chelator (PHM has an absolute requirement for copper) [89]. When wild-type *Chlamydomonas* were deflagellated in the presence of either inhibitor, reciliogenesis was significantly delayed, strongly suggesting that amidating activity is a key ciliogenic parameter. That reciliogenesis was delayed rather than completely inhibited likely reflects the observation that *Chlamydomonas* maintains sufficient components (potentially including enough amidated products) in the cell body to build two approximately half-length cilia without additional protein synthesis [90]. Third, *Chlamydomonas* cells expressing CrPAMΔCD, a mutant with functional catalytic cores that lacks ~ 75% of the CD, have enhanced enzyme activity as the truncated protein accumulates in the secretory pathway, and make full-length cilia that are completely motile [37]. Furthermore, insects only express separate PHM and PAL proteins [66], and while for many (e.g., *Drosophila*) the PAL protein also has an associated TMD/CD, recent database searches suggest that one insect order—the lepidoptera—express only soluble PAL lacking the TMD/CD again indicating the catalytic domains may be important. Although insects lack multiciliated epithelial cells, they still build ciliated motile sperm and use modified cilia for mechano- and chemo-sensation and in the chordotonal organ [91–93]. These experimental and bioinformatics observations suggest that the PAM CD may not play a fundamentally essential role in cilia formation and thus that the catalytic domains likely provide a key ciliogenic factor. The ability of PAM to amidate the C-termini of proteins (e.g., ubiquitin and monoclonal antibody heavy chains [94]), selected lipids [95, 96] and other metabolites, complicates the search for substrates. Site-directed mutants lacking PHM and/or PAL activity will be needed to definitively answer this important question.

## Models for the role of PAM in ciliogenesis

**Fig. 8:**
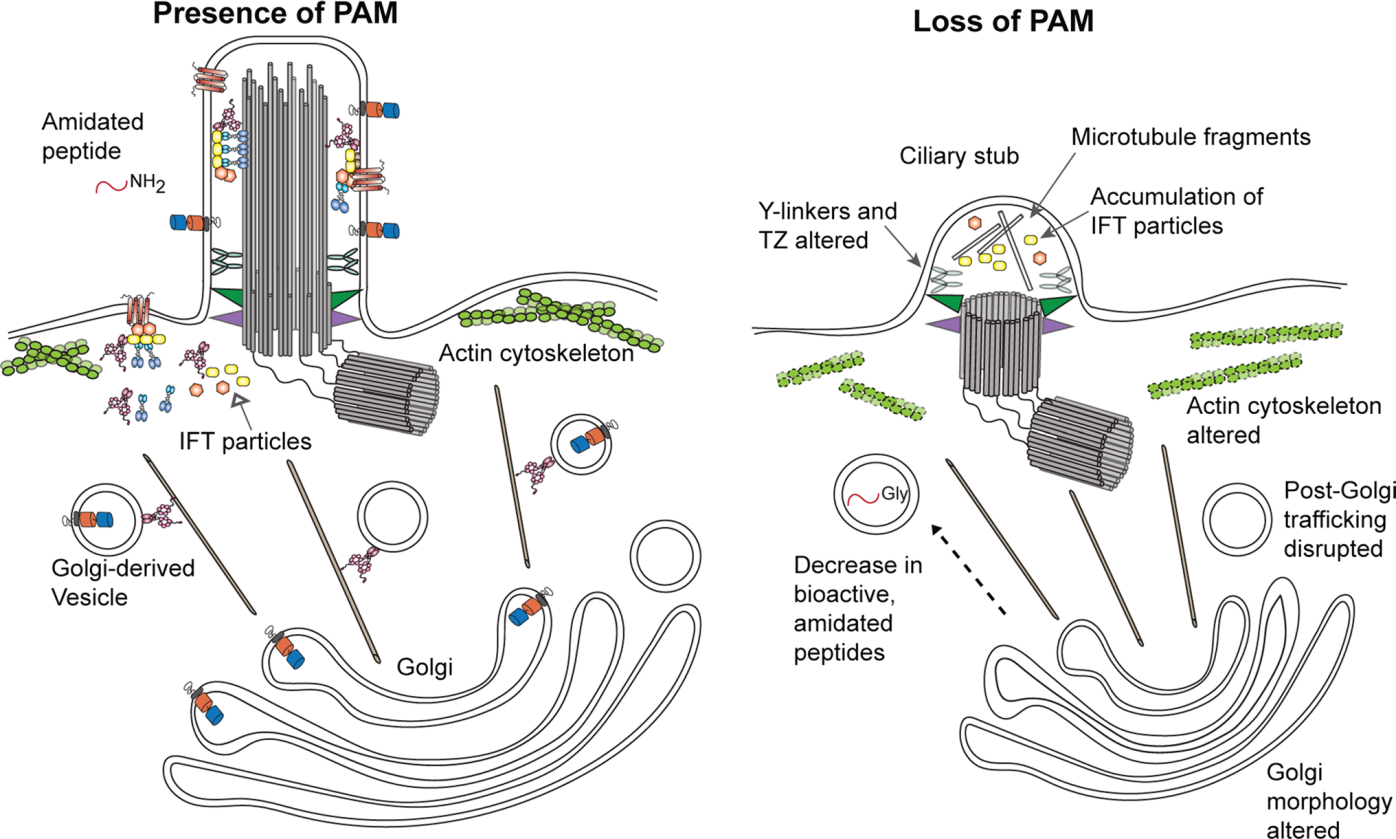
Model for PAM function during ciliogenesis in *Chlamydomonas.* Model depicting the general phenotypes observed in *Chlamydomonas* caused by the reduction of PAM expression. These include alterations in both Golgi morphology, which became more highly curved, and post-Golgi trafficking that may be a consequence of actin reorganization. In addition, transition zone architecture was disrupted and Y-links, which form a key component of the ciliary gate, were absent. Only short ciliary stubs formed; these accumulated IFT particle components and short microtubule fragments. Proteins shown are identified in the key to Fig. 3

The available evidence supports a species-specific role for amidating activity in promoting ciliogenesis. However, the identity of the substrate(s), product(s) and downstream target(s) important for this process remain unknown. In addition to a general lack of amidated products in the secretory pathway, PAM deficiency leads to several other distinct effects, each of which might also impact a different aspect of the ciliogenic pathway (Fig. 8). In *Chlamydomonas*, loss of PAM alters Golgi structure as the membrane stacks become more highly curved [37]. This may reflect a role for PAM in modifying or counteracting imposed membrane curvature, perhaps through its associations (either direct or via Rho-GEFs) with the actin cytoskeleton [38, 80]. Secretion of a subset of proteins is diminished when PAM levels are reduced [37]. Clathrin levels more than double in PAM-deficient cells, which may alter the availability of ciliary membrane lipid and/or protein components. Altered vesicular trafficking due to the loss of PAM might disrupt the balance between ciliary membrane incorporation and its loss through membrane budding and ectosome release from cilia. Changes in branched actin dynamics in PAM-deficient cilia might also cause membrane loss at a rate that cannot be fully replenished. Together these processes could result in the failure of new cilia to assemble normally, and lead to the gradual loss of existing cilia as their membrane is shed. There is also clear evidence that lack of PAM in *Chlamydomonas* affects formation of the TZ and disrupts or modifies the ciliary gate such that IFT components accumulate in the post-TZ stubs [37]. Potentially, this too might reflect altered ciliary membrane lipid content affecting TZ assembly. Interestingly, *Chlamydomonas* responds to the loss of PAM with a large increase in the level of two TZ proteins (CEP290 and NPHP4) and an IFT-A protein (IFT139) [37]; these proteins are all cytosolic and thus cannot be direct substrates for PAM as its enzymatic cores are located in the secretory pathway lumen. This suggests a more complex response to the lack of PAM leading to compromised ciliary exit and disrupted regulatory processes.

**Fig. 9:**
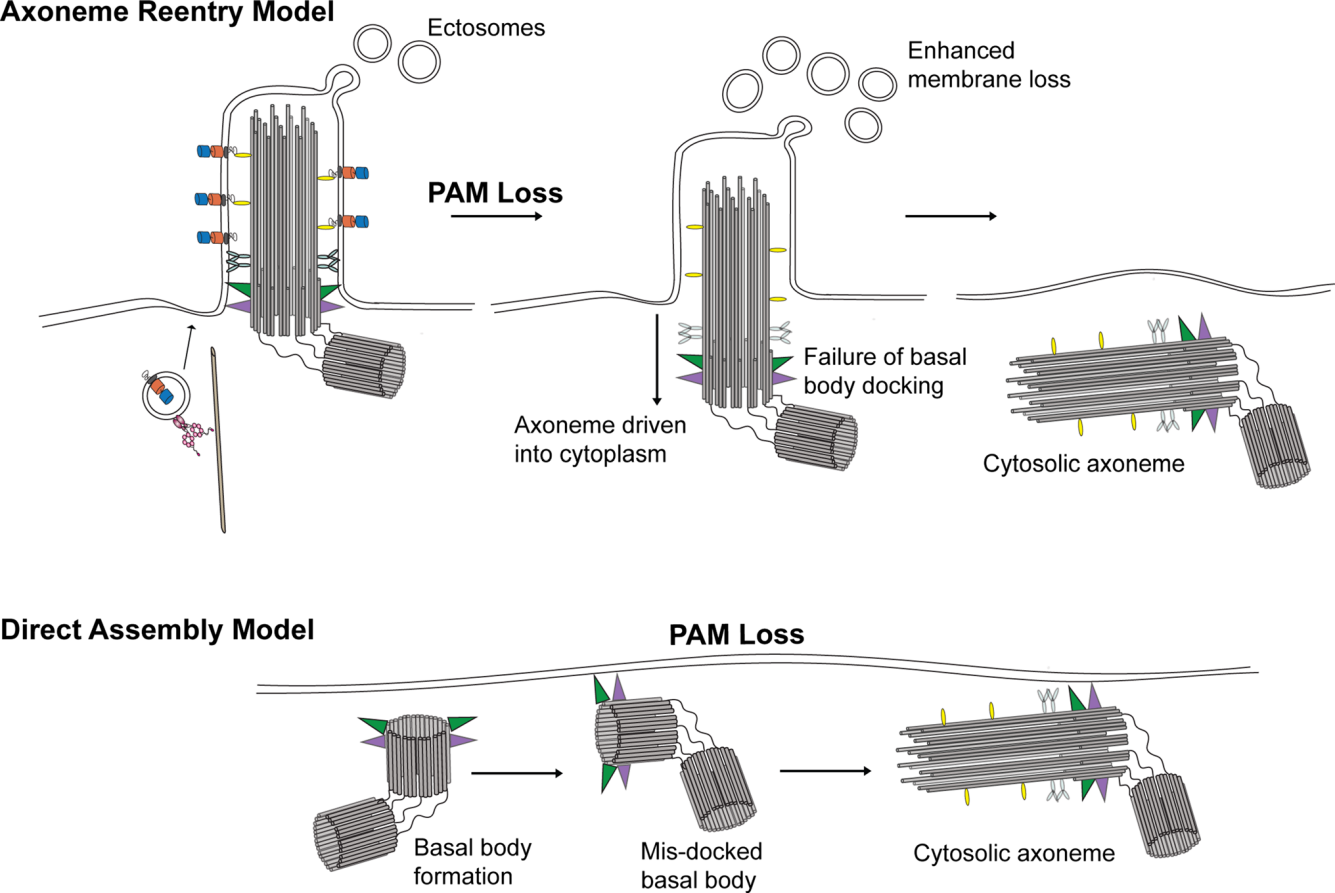
Models for formation of cytosolic axonemes. Two models for how axonemes might form in the cytoplasm of multiciliated cells following loss of PAM. In the “axoneme reentry” model, PAM loss leads to enhanced ciliary membrane loss and failure of basal body docking, and results in the preassembled axoneme being driven into the cytoplasm. Alternatively, in the “direct assembly” model, lack of PAM might lead to misoriented newly synthesized basal bodies at the plasma membrane, which could then act to template axoneme assembly from components concentrated in this peri-basal body region of cytoplasm

In multiciliated epithelial cells of both planaria (Fig. 7b) and zebrafish, loss of PAM leads to the presence of cytosolic membrane-less axonemes [37, 38]. There are two general mechanisms by which these might form (Fig. 9). In the “axoneme reentry” model, membrane-less axonemes would derive from previously assembled cilia that have experienced enhanced ciliary membrane loss and basal body undocking from the plasma membrane and have thus been driven back into the cytoplasm. This is essentially the reverse of exflagellation, the process used by *Plasmodium* to extrude a membrane-less axoneme assembled in the cytoplasm [97]. In the “direct assembly” model, defects in basal body docking after their initial formation might lead to the exposure of the axonemal assembly template in the cytoplasm near the plasma membrane where stores of ciliary components are localized, thereby allowing for axoneme formation in the cytoplasm. One observation supporting a specific defect in basal body docking is that in planaria, nearly all of the cytosolic axonemes were located very close to the plasma membrane and oriented in approximately the same direction, with the distal tip towards the front of the animal. This observation implies some type of directional signal that leads to a specific basal body orientation. The “direct assembly” model predicts that cytosolic axoneme assembly is an ancestral or default state. In the absence of compartmentalization within the ciliary membrane or proper regulation of the axoneme assembly properties of the basal body template (as mediated, for example, by CP110 [98]), cytosolic axoneme formation could occur spontaneously.

## What function(s) might ciliary localized PAM perform?

In addition to its role in cilia formation, PAM is also trafficked into these organelles; in *Chlamydomonas*, it becomes stably associated with the microtubular axoneme [36]. The topology of ciliary-PAM places its catalytic domains on the external face of the organelle. Although PHM has an acidic pH optimum, it retains some activity under neutral conditions; in the presence of adequate copper, ascorbate and molecular oxygen, cilia-localized PAM is expected to be catalytically active. Thus, ciliary PAM could act on glycine-extended proteins present in the extracellular environment or associated with the external face of the ciliary membrane, generating amidated products which might play a role in signaling or some other process. In situations where cilia interact, e.g., during mating in *Chlamydomonas*, ciliary PAM might function in *trans*, amidating substrates present on the surface of the interacting cilium. In *Chlamydomonas*, the ciliary membrane is the only membrane normally directly exposed to the environment (except when the cell wall is shed during the mating reaction). Local generation of amidated factors might play a key role in providing a sufficiently high concentration of modified products for productive downstream signaling. As PAM CD binds actin directly with sub-μM affinity [38], it is also possible that ciliary PAM alters the formation or dynamics of short branched actin filaments within cilia, a process which is thought to control primary ciliogenesis in animal cells [98, 99].

Intriguingly, PAM is tightly associated with the axoneme and in vitro, unlike most ciliary membrane proteins, PAM is only solubilized when demembranated cilia are treated with increased salt concentrations, as observed for several other axonemal substructures such as the dynein arms. This interaction might occur directly, through association of the intraciliary PAM CD with the axoneme, or indirectly, through an axoneme-associated component (which might even extend across the membrane into the extracellular space). Why is this unusual transmembrane protein tethering necessary? One possible explanation is that it provides a mechanism to avoid the general, non-specific or unregulated loss of membrane-associated PAM from the cilium—either in ectosomes budded from the ciliary membrane or from BBSome/IFT-mediated recycling back to the cell body. A further puzzle is the apparent 250–300 nm spacing for PAM observed by immunogold EM [36]; this spacing does not directly correlate with other known axonemal repeat distances.

## A phylogenetic conundrum—many organisms lacking PAM still build cilia

**Fig. 10:**
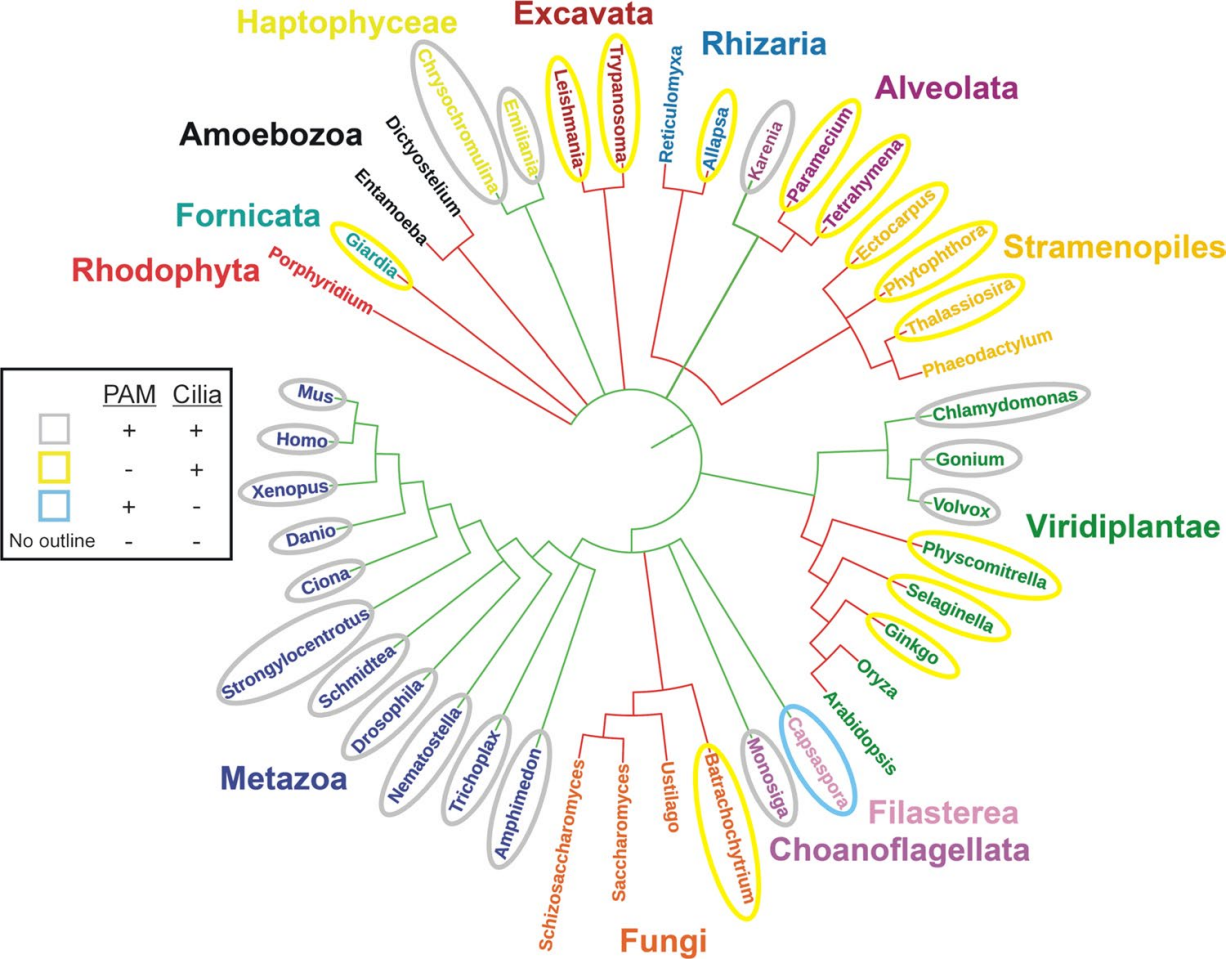
PAM expression and ciliogenesis throughout the eukaryotes. Phylogenetic tree illustrating members of the main eukaryotic groups. Connecting lines indicate the presence (green) or absence (red) of PAM (or PHM) in specific lineages. Organisms that have both PAM and cilia are outlined in grey; organisms that lack PAM but build cilia are highlighted in yellow. *Capsaspora*, which has PAM but lacks cilia, is indicated with a blue oval, and organisms that lack both PAM and cilia are not outlined. Although the precise branching of some deep phylogenetic divisions remains unresolved [123] and is not addressed here, currently available sequence data suggest that PAM has undergone multiple independent loss events in different lineages at least some members of which still retain cilia; e.g., in the fungi, plants, and alveolates. The initial plot was generated using PHYLOT [124] and manually modified and annotated

PAM and/or separate PHM and PAL modules are present throughout metazoans and the chlorophyte green algae; in addition to key roles in peptidergic signaling, in both these lineages PAM is involved in ciliogenesis. However, the pattern of PAM-like gene expression in other eukaryotic groups is more varied, suggesting that this enzyme has undergone multiple independent loss events during evolution and that in some organisms, ciliary assembly in the absence of PAM occurs (Fig. 10). For example, within the opisthokonts (which includes the metazoa, fungi, choanoflagellates and filasterea), PAM is missing throughout the fungi, even though the chytrids build flagellated zoospores. In contrast, although a previous bioinformatics analysis did not find evidence for PAM-related gene(s) in choanoflagellates, more recent database searches revealed a PHM-like sequence in *Monosiga brevicollis* (XP_001746677) and both PHM- (GGOY01024199) and PAL-like (GGOY01026207) sequences in *Salpingoeca urceolata*. Similarly, membrane-PAM is present in the filasterean *Capsaspora owczarzaki* (XP_004363913). A second clear instance of PAM loss occurs in the green plant lineage. Although PAM is found in ciliated unicellular and colonial chlorophyte algae (*Chlamydomonas*, *Gonium* and *Volvox*), it is missing in all land plants, including mosses and *Ginkgo*, which both have flagellated sperm cells.

Genomic studies reveal that numerous other ciliated groups, including excavates (e.g., *Trypanosoma*), stramenopiles (e.g., diatoms, oomycetes and brown algae), and fornicates (e.g., *Giardia*), also completely lack PAM. Intriguingly, a more complex pattern of loss is apparent in the ciliated alveolates. Some, such as *Tetrahymena* and *Paramecium*, do not encode PAM-like sequences. However, PHM- (EX872387) and PAL-like (EX872386) partial sequences were previously reported in another alveolate—the dinoflagellate *Karenia brevis* [66]. Furthermore, there are now two examples of PAM-like genes in ciliated haptophytes—unicellular organisms abundant in the marine phytoplankton; both *Emiliania huxleyi* and *Chrysochromulina* sp. express a soluble PHM + PAL (XP_005764558 and KOO21218, respectively). Haptophytes have calcite-based coccoliths forming an exoskeleton and two motile cilia. Furthermore, they assemble an additional thin microtubule-based cellular protrusion (the haptonema), and use bidirectional transport along this structure for food/prey retrieval [100] in a process that, at least superficially, is remarkably reminiscent of IFT-driven bead transport along *Chlamydomonas* cilia [101, 102]; haptonema may also play a sensory role. Together, these observations suggest that the presence/absence of PAM may define a fundamental dichotomy in the assembly and/or function of cilia and divide the ciliated eukaryotes into two broad groupings. Identification of genes expressed in ciliated organisms encoding PAM but missing in ciliated organisms lacking PAM may provide a path to revealing a unique subset of genes that affect or define this intriguing ciliogenic pathway.

A broad and diverse array of non-ciliated unicellular organisms (e.g., yeasts, amoebae, rhizarians such as *Reticulomyxa*, and the red alga *Cyanidioschyzon merolae*) lack PAM. However, that PAM likely plays non-cilia related roles in at least some unicellular organisms is evidenced by its retention in the filasterean *C. owczarzaki*, which appears to lack all cilia-specific genes [103]. Similarly, the planktonic pico-chlorophyte *Ostreococcus lucimarinus*, which is entirely missing any IFT machinery, also has PAM; this organism does not build cilia and retains only a single axonemal inner arm dynein that may have been repurposed for a non-ciliary role [104, 105]. Likewise, the presence of PHM sequences in both *Coccomyxa* sp. and *Chlorella variabilis* [66] further suggest a cilia-independent role for amidation in green algae; intriguingly, although both these organisms encode components of the outer dynein arm [106, 107], they lack most of the IFT system and neither has been observed to form cilia.

## Implications for cilia-based signaling and ciliopathies

Ciliopathies represent a broad group of multisystemic disorders that derive from defective cilia-based signaling and/or motile behavior [12, 17]. Signaling-associated phenotypes can include severe brain malformations (e.g., Joubert and Meckel Syndromes [108]), skeletal abnormalities (e.g., juvenile thoracic dystrophy and short rib polydactyly [16]), polycystic kidney disease (the most common genetic disorder in humans with an incidence of ~ 1/1000) [109], and other complex syndromes with multiple overlapping clinical features such as rod/cone dystrophy, mental retardation, obesity, anosmia, and insulin resistance (e.g., Bardet–Biedl syndrome [110]). Recent studies have revealed human genetic variants in PAM affecting insulin resistance [111], altered risk for diabetes [112], and hypertension with associated insulin resistance and altered low density lipoprotein levels [113]. As many ciliopathies include endocrine features such as obesity in their pathology, this raises the possibility that ciliary PAM defects contribute to these complex phenotypes.

Defects in motile cilia result in primary ciliary dyskinesia [114]. Phenotypes include inhibition of sperm motility, resulting in male infertility. More generally, defective ciliary motility compromises lung function as secreted mucus, which acts as a protectant, cannot be cleared, and cerebrospinal fluid flow in the brain ventricles is restricted, leading to hydrocephalus. In addition, left–right patterning is disrupted by cilia dysfunction at the embryonic node, resulting in situs inversus or heterotaxy. Lack of cilia-driven flow in the fallopian tubes, which is required to move oocytes to the uterus, causes female infertility. In these latter cases, it is the cilia-driven movement of fluid bathing the ciliated epithelium that is required for normal physiology. Although no obvious motile cilia phenotypes (such as laterality defects) have yet been described in PAM knockout mice, the zebrafish null mutants exhibit hydrocephalus and kidney-associated cysts, both of which can derive from defective ciliary motility in this organism.

## Conclusions

Recent studies revealed an unexpected role for the peptide amidating monooxygenase (PAM), a highly conserved copper-, molecular oxygen- and ascorbate-dependent secretory pathway enzyme, in building cilia. This connection has been observed in both vertebrates and algae, suggesting that it dates to the last common ancestor of eukaryotes and represents a fundamental feature of ciliogenesis. Current data support a role for amidating activity in forming the ciliary gate and regulation of the IFT system. Numerous studies have also revealed a connection between PAM and control of the actin cytoskeleton, and lead to the suggestion that PAM might play a central role in trafficking of membrane or other components to these cytoskeleton-based cellular extensions. Key issues for the future include identifying the PAM substrate(s) involved in ciliogenesis and determining the role and fate of ciliary-localized PAM and its potential amidated products.

## References

[1] Bloodgood RA (2010). Sensory reception is an attribute of both primary cilia and motile cilia. J Cell Sci.

[2] Marshall W, Basto R (2017). Cilia. Cold Spring Harbor perspectives in biology.

[3] Nager AR, Goldstein JS, Herranz-Pérez V, Portran D, Ye F, Garcia-Verdugo JM, Nachury MV (2017). An actin network dispatches ciliary gpcrs into extracellular vesicles to modulate signaling. Cell.

[4] Wood CR, Huang K, Diener DR, Rosenbaum JL (2013). The cilium secretes bioactive ectosomes. Curr Biol.

[5] Wang J, Silva M, Haas LA, Morsci NS, Nguyen KCQ, Hall DH, Barr MM (2014). *C. elegans* ciliated sensory neurons release extracellular vesicles that function in animal communication. Curr Biol.

[6] Davenport J, Yoder BK (2005). An incredible decade for the primary cilium: a new look at a once-forgotten organelle. Am J Physiol Ren Physiol.

[7] Pazour G, Witman G (2003). The vertebrate primary cilium is a sensory organelle. Curr Opin Cell Biol.

[8] Inglis PN, Boroevich KA, Leroux MR (2006). Piecing together a ciliome. Trends Genet.

[9] Sigg MA, Menchen T, Lee C, Johnson J, Jungnickel MK, Choksi SP, Garcia G, Busengdal H, Dougherty GW, Pennekamp P, Werner C, Rentzsch F, Florman HM, Krogan N, Wallingford JB, Omran H, Reiter JF (2017). Evolutionary proteomics uncovers ancient associations of cilia with signaling pathways. Dev Cell.

[10] van Dam TJP, Kennedy J, van der Lee R, de Vrieze E, Wunderlich KA, Rix S, Dougherty GW, Lambacher NJ, Li C, Jensen VL, Leroux MR, Hjeij R, Horn N, Texier Y, Wissinger Y, van Reeuwijk J, Wheway G, Knapp B, Scheel JF, Franco B, Mans DA, van Wijk E, Képès F, Slaats GG, Toedt G, Kremer H, Omran H, Szymanska K, Koutroumpas K, Ueffing M, Nguyen T-MT, Letteboer SJF, Oud MM, van Beersum SEC, Schmidts M, Beales PL, Lu Q, Giles RH, Szklarczyk R, Russell RB, Gibson TJ, Johnson CA, Blacque OE, Wolfrum U, Boldt K, Roepman R, Hernandez-Hernandez V, Huynen MA (2017). CiliaCarta: an integrated and validated compendium of ciliary genes. BioRxiv.

[11] Pertea M, Shumate A, Pertea G, Varabyou A, Chang Y-C, Madugundu AK, Pandey A, Salzberg S (2018). Thousands of large-scale RNA sequencing experiments yield a comprehensive new human gene list and reveal extensive transcriptional noise. BioRxiv.

[12] Reiter JF, Leroux MR (2017). Genes and molecular pathways underpinning ciliopathies. Nat Rev Mol Cell Biol.

[13] Ansley SJ, Badano JL, Blacque OE, Hill J, Hoskins BE, Leitch CC, Chul Kim J, Ross AJ, Eichers ER, Teslovich TM, Mah AK, Johnsen RC, Cavender JC, Alan Lewis R, Leroux MR, Beales PL, Katsanis N (2003). Basal body dysfunction is a likely cause of pleiotropic Bardet–Biedl syndrome. Nature.

[14] Doherty D (2009). Joubert syndrome: insights into brain development, cilium biology, and complex disease. Semin Pediatr Neurol.

[15] Hildebrandt F, Benzing T, Katsanis N (2011). Ciliopathies. N Engl J Med.

[16] Schmidts M, Mitchison H, King SM (2018). Severe skeletal abnormalities caused by defects in retrograde intraflagellar transport. Dyneins: structure, biology and disease—volume 2—dynein mechanics, dysfunction and disease.

[17] Fliegauf M, Benzing T, Omran H (2007). When cilia go bad: cilia defects and ciliopathies. Nat Rev Mol Cell Biol.

[18] Gonçalves J, Pelletier L (2017). The ciliary transition zone: finding the pieces and assembling the gate. Mol Cells.

[19] Diener DR, Lupetti P, Rosenbaum JL (2015). Proteomic analysis of isolated ciliary transition zones reveals the presence of ESCRT proteins. Curr Biol.

[20] Lienkamp S, Ganner A, Walz G (2012). Inversin, Wnt signaling and primary cilia. Differentiation.

[21] Wallingford JB (2006). Planar cell polarity, ciliogenesis and neural tube defects. Hum Mol Genet.

[22] Bangs F, Anderson KV (2016). Primary cilia and mammalian hedgehog signaling. Cold Spring Harb Perspect Biol.

[23] Huangfu D, Liu A, Rakeman AS, Murcia NS, Niswander L, Anderson KV (2003). Hedgehog signalling in the mouse requires intraflagellar transport proteins. Nature.

[24] Händel M, Schulz S, Stanarius A, Schreff M, Erdtmann-Vourliotis M, Schmidt H, Wolf G, Höllt V (1999). Selective targeting of somatostatin receptor 3 to neuronal cilia. Neuroscience.

[25] Koemeter-Cox AI, Sherwood TW, Green JA, Steiner RA, Berbari NF, Yoder BK, Kauffman AS, Monsma PC, Brown A, Askwith CC, Mykytyn K (2014). Primary cilia enhance kisspeptin receptor signaling on gonadotropin-releasing hormone neurons. Proc Natl Acad Sci USA.

[26] Johnson NT, Villalon M, Royce FH, Hard R, Verdugo P (1991). Autoregulation of beat frequency in respiratory ciliated cells. Demonstration by viscous loading. Am Rev Respir Dis.

[27] Rompolas P, Patel-King RS, King SM (2012). Association of Lis1 with outer arm dynein is modulated in response to alterations in flagellar motility. Mol Biol Cell.

[28] Shah AS, Ben-Shahar Y, Moninger TO, Kline JN, Welsh MJ (2009). Motile cilia of human airway epithelia are chemosensory. Science.

[29] Pan J, Snell WJ (2000). Signal transduction during fertilization in the unicellular green alga, *Chlamydomonas*. Curr Opin Microbiol.

[30] Cantagrel V, Silhavy JL, Bielas SL, Swistun D, Marsh SE, Bertrand JY, Audollent S, Attié-Bitach T, Holden KR, Dobyns WB, Traver D, Al-Gazali L, Ali BR, Lindner TH, Caspary T, Otto EA, Hildebrandt F, Glass IA, Logan CV, Johnson CA, Bennett C, Brancati F, Valente EM, Woods CG, Gleeson JG (2008). Mutations in the cilia gene ARL13B Lead to the classical form of Joubert syndrome. Am J Hum Genet.

[31] Asante D, MacCarthy-Morrogh L, Townley AK, Weiss MA, Katayama K, Palmer KJ, Suzuki H, Westlake CJ, Stephens DJ (2013). A role for the Golgi matrix protein giantin in ciliogenesis through control of the localization of dynein-2. J Cell Sci.

[32] Marszalek JR, Goldstein LS (2000). Understanding the functions of kinesin-II. Biochim Biophys Acta.

[33] Kim J, Jo H, Hong H, Kim MH, Kim JM, Lee JK, Heo WD, Kim J (2015). Actin remodelling factors control ciliogenesis by regulating YAP/TAZ activity and vesicle trafficking. Nat Commun.

[34] Avasthi P, Marshall WF (2012). Stages of ciliogenesis and regulation of ciliary length. Differentiation.

[35] Verghese E, Zhuang J, Saiti D, Ricardo Sharon D, Deane James A (2013). In vitro investigation of renal epithelial injury suggests that primary cilium length is regulated by hypoxia-inducible mechanisms. Cell Biol Int.

[36] Kumar D, Blaby-Haas CE, Merchant SS, Mains RE, King SM, Eipper BA (2016). Early eukaryotic origins for cilia-associated bioactive peptide amidating activity. J Cell Sci.

[37] Kumar D, Strenkert D, Patel-King RS, Leonard MT, Merchant SS, Mains RE, King SM, Eipper BA (2017). A bioactive peptide amidating enzyme is required for ciliogenesis. eLife.

[38] Kumar D, Thomason RT, Yankova M, Gitlin JD, Mains RE, Eipper BA, King SM (2018). Microvillar and ciliary defects in zebrafish lacking an actin-binding bioactive peptide amidating enzyme. Sci Rep.

[39] Sorokin SP (1968). Reconstructions of centriole formation and ciliogenesis in mammalian lungs. J Cell Sci.

[40] Dawe HR, Farr H, Gull K (2007). Centriole/basal body morphogenesis and migration during ciliogenesis in animal cells. J Cell Sci.

[41] Cole DG, Diener DR, Himelblau AL, Beech PL, Fuster JC, Rosenbaum JL (1998). *Chlamydomonas* kinesin-II-dependent intraflagellar transport (IFT): IFT particles contain proteins required for ciliary assembly in *Caenorhabditis elegans* sensory neurons. J Cell Biol.

[42] Ou G, Koga M, Blacque OE, Murayama T, Ohshima Y, Schafer JC, Li C, Yoder BK, Leroux MR, Scholey JM (2007). Sensory ciliogenesis in *Caenorhabditis elegans*: assignment of IFT components into distinct modules based on transport and phenotypic profiles. Mol Biol Cell.

[43] Rosenbaum JL, Witman GB (2002). Intraflagellar transport. Nat Rev Mol Cell Biol.

[44] Blacque OE, Li C, Inglis PN, Esmail MA, Ou G, Mah AK, Baillie DL, Scholey JM, Leroux MR (2006). The WD repeat-containing protein IFTA-1 is required for retrograde intraflagellar transport. Mol Biol Cell.

[45] Chien A, Shih SM, Bower R, Tritschler D, Porter ME, Yildiz A (2017). Dynamics of the IFT machinery at the ciliary tip. eLife.

[46] Luo W, Ruba A, Takao D, Zweifel LP, Lim RYH, Verhey KJ, Yang W (2017). Axonemal lumen dominates cytosolic protein diffusion inside the primary cilium. Sci Rep.

[47] Garcia G, Raleigh DR, Reiter JF (2018). How the ciliary membrane is organized inside-out to communicate outside-in. Curr Biol.

[48] Jin H, White SR, Shida T, Schulz S, Aguiar M, Gygi SP, Bazan JF, Nachury MV (2010). The conserved Bardet–Biedl syndrome proteins assemble a coat that traffics membrane proteins to cilia. Cell.

[49] Lechtreck KF, Johnson EC, Sakai T, Cochran D, Ballif BA, Rush J, Pazour GJ, Ikebe M, Witman GB (2009). The C*hlamydomonas reinhardtii* BBSome is an IFT cargo required for export of specific signaling proteins from flagella. J Cell Biol.

[50] Nachury MV, Loktev AV, Zhang Q, Westlake CJ, Peranen J, Merdes A, Slusarski DC, Scheller RH, Bazan JF, Sheffield VC, Jackson PK (2007). A core complex of BBS proteins cooperates with the GTPase Rab8 to promote ciliary membrane biogenesis. Cell.

[51] Nachury MV, Seeley ES, Jin H (2010). Trafficking to the ciliary membrane: how to get across the periciliary diffusion barrier?. Annu Rev Cell Dev Biol.

[52] Liu P, Lechtreck KF (2018). The Bardet–Biedl syndrome protein complex is an adapter expanding the cargo range of intraflagellar transport trains for ciliary export. Proc Natl Acad Sci USA.

[53] Bhogaraju S, Cajanek L, Fort C, Blisnick T, Weber K, Taschner M, Mizuno N, Lamla S, Bastin P, Nigg EA, Lorentzen E (2013). Molecular basis of tubulin transport within the cilium by IFT74 and IFT81. Science.

[54] Taschner M, Mourão A, Awasthi M, Basquin J, Lorentzen E (2017). Structural basis of outer dynein arm intraflagellar transport by the transport adaptor protein ODA16 and the intraflagellar transport protein IFT46. J Biol Chem.

[55] Wood CR, Wang Z, Diener D, Zones JM, Rosenbaum J, Umen JG (2012). IFT proteins accumulate during cell division and localize to the cleavage furrow in *Chlamydomonas*. PLoS One.

[56] Delaval B, Bright A, Lawson ND, Doxsey S (2011). The cilia protein IFT88 is required for spindle orientation in mitosis. Nat Cell Biol.

[57] Bizet AA, Becker-Heck A, Ryan R, Weber K, Filhol E, Krug P, Halbritter J, Delous M, Lasbennes M-C, Linghu B, Oakeley EJ, Zarhrate M, Nitschké P, Garfa-Traore M, Serluca F, Yang F, Bouwmeester T, Pinson L, Cassuto E, Dubot P, Elshakhs NAS, Sahel JA, Salomon R, Drummond IA, Gubler M-C, Antignac C, Chibout S, Szustakowski JD, Hildebrandt F, Lorentzen E, Sailer AW, Benmerah A, Saint-Mezard P, Saunier S (2015). Mutations in TRAF3IP1/IFT54 reveal a new role for IFT proteins in microtubule stabilization. Nat Commun.

[58] Kumar D, Mains RE, Eipper BA (2016). 60 years of POMC: from POMC and α-MSH to PAM, molecular oxygen, copper, and vitamin C. J Mol Endocrinol.

[59] Ward G, Brokaw C, Garbers D, Vacquier V (1985). Chemotaxis of *Arbacia punctulata* spermatozoa to Resact, a peptide from the egg jelly layer. J Cell Biol.

[60] Violette A, Biass D, Dutertre S, Koua D, Piquemal D, Pierrat F, Stöcklin R, Favreau P (2012). Large-scale discovery of conopeptides and conoproteins in the injectable venom of a fish-hunting cone snail using a combined proteomic and transcriptomic approach. J Proteom.

[61] Ul-Hasan S, Burgess DM, Gajewiak J, Li Q, Hu H, Yandell M, Olivera BM, Bandyopadhyay PK (2013). Characterization of the peptidylglycine α-amidating monooxygenase (PAM) from the venom ducts of neogastropods, *Conus bullatus* and *Conus geographus*. Toxicon.

[62] Robinson SD, Mueller A, Clayton D, Starobova H, Hamilton BR, Payne RJ, Vetter I, King GF, Undheim EAB (2018). A comprehensive portrait of the venom of the giant red bull ant, *Myrmecia gulosa*, reveals a hyperdiverse hymenopteran toxin gene family. Sci Adv.

[63] Grishin E (2001). Polypeptide neurotoxins from spider venoms. Eur J Biochem.

[64] Czyzyk TA, Ning Y, Hsu M-S, Peng B, Mains RE, Eipper BA, Pintar JE (2005). Deletion of peptide amidation enzymatic activity leads to edema and embryonic lethality in the mouse. Dev Biol.

[65] Jiang N, Kolhekar AS, Jacobs PS, Mains RE, Eipper BA, Taghert PH (2000). PHM is required for normal developmental transitions and for biosynthesis of secretory peptides in *Drosophila*. Dev Biol.

[66] Attenborough RMF, Hayward DC, Kitahara MV, Miller DJ, Ball EE (2012). A “neural” enzyme in nonbilaterian animals and algae: preneural origins for peptidylglycine α-amidating monooxygenase. Mol Biol Evol.

[67] Bäck N, Kanerva K, Kurutihalli V, Yanik A, Ikonen E, Mains RE, Eipper BA (2017). The endocytic pathways of a secretory granule membrane protein in HEK293 cells: PAM and EGF traverse a dynamic multivesicular body network together. Eur J Cell Biol.

[68] Gonzalez-Begne M, Lu B, Han X, Hagen FK, Hand AR, Melvin JE, Yates JR (2009). Proteomic analysis of human parotid gland exosomes by multidimensional protein identification technology (MudPIT). J Proteome Res.

[69] Minciacchi VR, Freeman MR, Di Vizio D (2015). Extracellular vesicles in cancer: exosomes, microvesicles and the emerging role of large oncosomes. Semin Cell Dev Biol.

[70] Rajagopal C, Stone KL, Mains RE, Eipper BA (2010). Secretion stimulates intramembrane proteolysis of a secretory granule membrane enzyme. J Biol Chem.

[71] Mains RE, Blaby-Haas CE, Rheaume BA, Eipper BA (2018). Changes in corticotrope gene expression upon increased expression of peptidylglycine α-amidating monooxygenase. Endocrinol.

[72] Hsiao Y-C, Tuz K, Ferland RJ (2012). Trafficking in and to the primary cilium. Cilia.

[73] Emmer BT, Maric D, Engman DM (2010). Molecular mechanisms of protein and lipid targeting to ciliary membranes. J Cell Sci.

[74] Boehlke C, Bashkurov M, Buescher A, Krick T, John A-K, Nitschke R, Walz G, Kuehn EW (2010). Differential role of Rab proteins in ciliary trafficking: Rab23 regulates smoothened levels. J Cell Sci.

[75] Lu L, Madugula V (2018). Mechanisms of ciliary targeting: entering importins and Rabs. Cell Mol Life Sci.

[76] Huang K, Diener DR, Mitchell A, Pazour GJ, Witman GB, Rosenbaum JL (2007). Function and dynamics of PKD2 in *Chlamydomonas reinhardtii* flagella. J Cell Biol.

[77] Pan J, You Y, Huang T, Brody SL (2007). RhoA-mediated apical actin enrichment is required for ciliogenesis and promoted by Foxj1. J Cell Sci.

[78] Phua SC, Chiba S, Suzuki M, Su E, Roberson EC, Pusapati GV, Setou M, Rohatgi R, Reiter JF, Ikegami K, Inoue T (2017). Dynamic remodeling of membrane composition drives cell cycle through primary cilia excision. Cell.

[79] Ciccotosto GD, Schiller MR, Eipper BA, Mains RE (1999). Induction of integral membrane PAM expression in AtT-20 cells alters the storage and trafficking of POMC and PC1. J Cell Biol.

[80] Alam MR, Johnson RC, Darlington DN, Hand TA, Mains RE, Eipper BA (1997). Kalirin, a cytosolic protein with spectrin-like and GDP/GTP exchange factor-like domains that interacts with peptidylglycine α-amidating monooxygenase, an integral membrane peptide-processing enzyme. J Biol Chem.

[81] Burridge K, Wennerberg K (2004). Rho and Rac take center stage. Cell.

[82] Kato-Minoura T, Uryu S, Hirono M, Kamiya R (1998). Highly divergent actin expressed in a *Chlamydomonas* mutant lacking the conventional actin gene. Biochem Biophys Res Commun.

[83] Lee MT, Bonneau AR, Takacs CM, Bazzini AA, DiVito KR, Fleming ES, Giraldez AJ (2013). Nanog, Pou5f1 and SoxB1 activate zygotic gene expression during the maternal-to-zygotic transition. Nature.

[84] Reiter JF, Blacque OE, Leroux MR (2012). The base of the cilium: roles for transition fibres and the transition zone in ciliary formation, maintenance and compartmentalization. EMBO Rep.

[85] Craige B, Tsao C-C, Diener DR, Hou Y, Lechtreck K-F, Rosenbaum JL, Witman GB (2010). CEP290 tethers flagellar transition zone microtubules to the membrane and regulates flagellar protein content. J Cell Biol.

[86] Choi SY, Chacon-Heszele MF, Huang L, McKenna S, Wilson FP, Zuo X, Lipschutz JH (2013). Cdc42 deficiency causes ciliary abnormalities and cystic kidneys. J Am Soc Nephrol.

[87] Rompolas P, Patel-King RS, King SM (2010). An outer arm dynein conformational switch is required for metachronal synchrony of motile cilia in planaria. Mol Biol Cell.

[88] Katopodis AG, May SW (1990). Novel substrates and inhibitors of peptidylglycine α-amidating monooxygenase. Biochemistry.

[89] Mendelsohn BA, Yin C, Johnson SL, Wilm TP, Solnica-Krezel L, Gitlin JD (2006). Atp7a determines a hierarchy of copper metabolism essential for notochord development. Cell Metab.

[90] Rosenbaum JL, Moulder JE, Ringo DL (1969). Flagellar elongation and shortening in *Chlamydomonas*. The use of cycloheximide and colchicine to study the synthesis and assembly of flagellar proteins. J Cell Biol.

[91] Mencarelli C, Lupetti P, Dallai R (2008). New insights into the cell biology of insect axonemes. Int Rev Cell Mol Biol.

[92] Mancini K, Dolder H (2001). Ultrastructure of apyrene and eupyrene spermatozoa from the seminal vesicle of *Euptoieta hegesia* (Lepidoptera: Nymphalidae). Tissue Cell.

[93] Yack J, Roots B (1992). The metathoracic wing-hinge chordotonal organ of an atympanate moth, *Actias luna* (Lepidoptera, Saturniidae): a light- and electron-microscopic study. Cell Tissue Res.

[94] Liu H, Ponniah G, Zhang H-M, Nowak C, Neill A, Gonzalez-Lopez N, Patel R, Cheng G, Kita AZ, Andrien B (2014). In vitro and in vivo modifications of recombinant and human IgG antibodies. mAbs.

[95] Jeffries KA, Dempsey DR, Farrell EK, Anderson RL, Garbade GJ, Gurina TS, Gruhonjic I, Gunderson CA, Merkler DJ (2016). Glycine *N*-acyltransferase-like 3 is responsible for long-chain *N*-acylglycine formation in N18TG2 cells. J Lipid Res.

[96] Merkler DJ, Merkler KA, Stern W, Fleming FF (1996). Fatty acid amide biosynthesis: a possible new role for peptidylglycine α-amidating enzyme and acyl-coenzyme A: glycine *N*-acyltransferase. Arch Biochem Biophys.

[97] Avidor-Reiss T, Leroux Michel R (2015). Shared and distinct mechanisms of compartmentalized and cytosolic ciliogenesis. Curr Biol.

[98] Cao J, Shen Y, Zhu L, Xu Y, Zhou Y, Wu Z, Li Y, Yan X, Zhu X (2012). miR-129-3p controls cilia assembly by regulating CP110 and actin dynamics. Nat Cell Biol.

[99] Yan X, Zhu X (2012). Branched F-actin as a negative regulator of cilia formation. Expl Cell Res.

[100] Dölger J, Nielsen LT, Kiørboe T, Andersen A (2017). Swimming and feeding of mixotrophic biflagellates. Sci Rep.

[101] Hoffman JL, Goodenough UW (1980). Experimental dissection of flagellar surface motility in *Chlamydomonas*. J Cell Biol.

[102] Shih SM, Engel BD, Kocabas F, Bilyard T, Gennerich A, Marshall WF, Yildiz A (2013). Intraflagellar transport drives flagellar surface motility. eLife.

[103] Suga H, Chen Z, de Mendoza A, Sebé-Pedrós A, Brown MW, Kramer E, Carr M, Kerner P, Vervoort M, Sánchez-Pons N, Torruella G, Derelle R, Manning G, Lang BF, Russ C, Haas BJ, Roger AJ, Nusbaum C, Ruiz-Trillo I (2013). The *Capsaspora* genome reveals a complex unicellular prehistory of animals. Nat Commun.

[104] Palenik B, Grimwood J, Aerts A, Rouzé P, Salamov A, Putnam N, Dupont C, Jorgensen R, Derelle E, Rombauts S, Zhou K, Otillar R, Merchant SS, Podell S, Gaasterland T, Napoli C, Gendler K, Manuell A, Tai V, Vallon O, Piganeau G, Jancek S, Heijde M, Jabbari K, Bowler C, Lohr M, Robbens S, Werner G, Dubchak I, Pazour GJ, Ren Q, Paulsen I, Delwiche C, Schmutz J, Rokhsar D, Van de Peer Y, Moreau H, Grigoriev IV (2007). The tiny eukaryote *Ostreococcus* provides genomic insights into the paradox of plankton speciation. Proc Natl Acad Sci USA.

[105] Wickstead B, Gull K (2007). Dyneins across eukaryotes: a comparative genomic analysis. Traffic.

[106] Blanc G, Agarkova I, Grimwood J, Kuo A, Brueggeman A, Dunigan DD, Gurnon J, Ladunga I, Lindquist E, Lucas S, Pangilinan J, Pröschold T, Salamov A, Schmutz J, Weeks D, Yamada T, Lomsadze A, Borodovsky M, Claverie J-M, Grigoriev IV, Van Etten JL (2012). The genome of the polar eukaryotic microalga *Coccomyxa subellipsoidea* reveals traits of cold adaptation. Genome Biol.

[107] Blanc G, Duncan G, Agarkova I, Borodovsky M, Gurnon J, Kuo A, Lindquist E, Lucas S, Pangilinan J, Polle J, Salamov A, Terry A, Yamada T, Dunigan DD, Grigoriev IV, Claverie J-M, Van Etten JL (2010). The *Chlorella variabilis* NC64A genome reveals adaptation to photosymbiosis, coevolution with viruses, and cryptic sex. Plant Cell.

[108] Louie C, Gleeson J (2005). Genetic basis of Joubert syndrome and related disorders of cerebellar development. Hum Mol Genet.

[109] Pazour GJ, San Agustin JT, Follit JA, Rosenbaum JL, Witman GB (2002). Polycystin-2 localizes to kidney cilia and the ciliary level is elevated in orpk mice with polycystic kidney disease. Curr Biol.

[110] Li JB, Gerdes JM, Haycraft CJ, Fan Y, Teslovich TM, May-Simera H, Li H, Blacque OE, Li L, Leitch CC, Lewis RA, Green JS, Parfrey PS, Leroux MR, Davidson WS, Beales PL, Guay-Woodford LM, Yoder BK, Stormo GD, Katsanis N, Dutcher SK (2004). Comparative genomics identifies a flagellar and basal body proteome that includes the BBS5 human disease gene. Cell.

[111] Huyghe JR, Jackson AU, Fogarty MP, Buchkovich ML, Stančáková A, Stringham HM, Sim X, Yang L, Fuchsberger C, Cederberg H, Chines PS, Teslovich TM, Romm JM, Ling H, McMullen I, Ingersoll R, Pugh EW, Doheny KF, Neale BM, Daly MJ, Kuusisto J, Scott LJ, Kang HM, Collins FS, Abecasis GR, Watanabe RM, Boehnke M, Laakso M, Mohlke KL (2012). Exome array analysis identifies new loci and low-frequency variants influencing insulin processing and secretion. Nat Genet.

[112] Steinthorsdottir V, Thorleifsson G, Sulem P, Helgason H, Grarup N, Sigurdsson A, Helgadottir HT, Johannsdottir H, Magnusson OT, Gudjonsson SA, Justesen JM, Harder MN, Jørgensen ME, Christensen C, Brandslund I, Sandbæk A, Lauritzen T, Vestergaard H, Linneberg A, Jørgensen T, Hansen T, Daneshpour MS, Fallah M-S, Hreidarsson AB, Sigurdsson G, Azizi F, Benediktsson R, Masson G, Helgason A, Kong A, Gudbjartsson DF, Pedersen O, Thorsteinsdottir U, Stefansson K (2014). Identification of low-frequency and rare sequence variants associated with elevated or reduced risk of type 2 diabetes. Nat Genet.

[113] Yoo HJ, Kim M, Kim M, Chae JS, Lee S-H, Lee JH (2017). The peptidylglycine α-amidating monooxygenase (PAM) gene rs13175330 A>G polymorphism is associated with hypertension in a Korean population. Hum Genom.

[114] Loges N, Omran H, King SM (2018). Dynein dysfunction as a cause of primary ciliary dyskinesia. Dyneins: structure, biology and disease—volume 2—dynein mechanics, dysfunction and disease.

[115] Kumar D (2017) Control of ciliogenesis by an evolutionarily ancient peptide amidating enzyme. Ph.D. thesis, University of Connecticut

[116] Mijalkovic J, Prevo B, Oswald F, Mangeol P, Peterman EJG (2017). Ensemble and single-molecule dynamics of IFT dynein in *Caenorhabditis elegans* cilia. Nat Commun.

[117] Prevo B, Mangeol P, Oswald F, Scholey JM, Peterman EJG (2015). Functional differentiation of cooperating kinesin-2 motors orchestrates cargo import and transport in *C*. *elegans* cilia. Nat Cell Biol.

[118] Williams CL, McIntyre JC, Norris SR, Jenkins PM, Zhang L, Pei Q, Verhey K, Martens JR (2014). Direct evidence for BBSome-associated intraflagellar transport reveals distinct properties of native mammalian cilia. Nat Commun.

[119] Wingfield JL, Mengoni I, Bomberger H, Jiang Y-Y, Walsh JD, Brown JM, Picariello T, Cochran DA, Zhu B, Pan J, Eggenschwiler J, Gaertig J, Witman GB, Kner P, Lechtreck K (2017). IFT trains in different stages of assembly queue at the ciliary base for consecutive release into the cilium. eLife.

[120] Kumar D, King SM (2017). Trainspotting in a cilium. eLife.

[121] Prigge ST, Kolhekar AS, Eipper BA, Mains RE, Amzel LM (1997). Amidation of bioactive peptides: the structure of peptidylglycine α-hydroxylating monooxygenase. Science.

[122] Chufán EE, De M, Eipper BA, Mains RE, Amzel LM (2009). Amidation of bioactive peptides: the structure of the lyase domain of the amidating enzyme. Structure.

[123] Burki F (2014). The eukaryotic tree of life from a global phylogenomic perspective. Cold Spring Harb Perspect Biol.

[124] Letunic I, Bork P (2016). Interactive tree of life (iTOL) v3: an online tool for the display and annotation of phylogenetic and other trees. Nucleic Acids Res.

